# Influence of thermal and mechanical properties on surface integrity in CNC turning across multiple engineering materials

**DOI:** 10.1038/s41598-026-41648-3

**Published:** 2026-04-20

**Authors:** Mohammad S. Alsoufi, Saleh A. Bawazeer

**Affiliations:** Department of Mechanical Engineering, College of Engineering and Architecture, Umm Al- Qura University, Makkah, 21955 Kingdom of Saudi Arabia

**Keywords:** CNC turning, Hardness, Machining optimization, Surface roughness, Thermal conductivity, Waviness, Engineering, Materials science

## Abstract

Surface integrity is crucial in CNC machining, as it significantly impacts dimensional accuracy, fatigue life, and overall functional performance. This study examines the impact of thermal conductivity and hardness on surface roughness and waviness in five materials: Aluminum Alloy 6061, Brass C26000, Bronze C51000, Carbon Steel 1020 (annealed), and Stainless Steel 304 (annealed). All were machined under identical dry turning conditions to isolate material effects. The novelty lies in the combined analysis of roughness and waviness metrics, ISO-based profile ratios (*R*_q_/*R*_a_, *R*_t_/*R*_z_, *W*_q_/*W*_a_, *W*_t_/*W*_z_) that benchmark surface uniformity, and statistical shape descriptors (skewness, kurtosis) that indicate whether lubricant-retaining valleys or stress-concentrating asperities dominate surfaces, thereby extending beyond existing multi-material studies that primarily rely on mean roughness comparisons under identical cutting parameters. These metrics provide functional insights: negative skewness enhances tribological performance, while high kurtosis highlights asperities linked to fatigue crack initiation. Results show Stainless Steel 304 achieved the smoothest finish (*R*_a_ ≈ 0.9 μm, *W*_a_ ≈ 0.6 μm), while Carbon Steel 1020 produced the roughest (*R*_a_ ≈ 4.1 μm). A ± 10% change in thermal conductivity consistently shifted *R*_a_ by 5.8–6.3%. This study introduces a function-oriented, metrology-informed framework that translates surface descriptors into predictive indicators of wear resistance, fatigue performance, and dimensional reliability in high-precision manufacturing.

## Introduction

Surface integrity plays a crucial role in CNC machining, directly influencing the functional performance, durability, and reliability of machined components^1^. It encompasses multiple parameters, including surface roughness and surface waviness, which define the topographical characteristics of a machined surface and significantly impact friction, wear resistance, fatigue life, and adhesion properties^2^. Surface roughness (*R*_a_) captures fine-scale tool marks and is directly linked to tribological performance, influencing friction, lubrication, and fatigue crack initiation. For example, lower *R*_a_ improves bearing life and reduces wear in aerospace engine components, as well as controls tissue compatibility in biomedical implants. In contrast, surface waviness (*W*_a_) represents long-wavelength undulations caused by vibrations or tool deflection and has a distinct impact: excessive waviness can compromise gasket sealing effectiveness, impair optical alignment in precision assemblies, or induce vibration and noise in automotive parts. These examples demonstrate that roughness and waviness operate at different functional scales, and their combined evaluation provides a comprehensive picture of surface integrity, which is critical to high-performance applications^3^.

A deeper understanding of these parameters is essential in precision manufacturing, where minor variations in surface integrity can critically affect product functionality. Material properties, particularly thermal conductivity and hardness, are key determinants of surface integrity^4^. Thermal conductivity affects heat dissipation during machining, where low-thermal-conductivity materials, such as stainless steel 304 annealed, can lead to heat accumulation, accelerating tool wear, and degrading surface quality^5^. Conversely, high-thermal-conductivity materials like aluminum alloy 6061 efficiently dissipate heat, maintaining stable machining conditions and achieving smoother surfaces^6^. Hardness influences cutting resistance; harder materials tend to generate higher cutting forces, increasing surface roughness and surface waviness due to tool wear and material deformation^7^. These interactions highlight the importance of selecting optimal machining parameters based on material characteristics.

Although extensive research has been conducted on machining dynamics, a critical gap remains in multi-material comparative studies linking thermal conductivity and hardness to both surface roughness and surface waviness outcomes in CNC turning operations^8^. Most studies focus on single-material investigations, making it difficult to generalize their findings across various engineering alloys^9^. Additionally, previous research has primarily emphasized surface roughness, often neglecting the broader surface waviness, which can equally impact component performance in applications such as the aerospace, automotive, and biomedical industries^10^.

Recent work by Alsoufi and Elsayed emphasized the impact of measurement direction on surface roughness in fused deposition modeling (FDM), showing that roughness values vary with measurement orientation^11^. This highlights the importance of comprehensively assessing surface integrity, considering both measurement techniques and machining conditions. Similarly, Alsoufi et al. compared abrasive waterjet (AWJ) and laser beam (LB) machining and found significant differences in surface roughness and hardness between these cutting technologies^12^. Their research reinforces the need for multi-material studies that integrate mechanical and thermal properties in CNC machining.

This study seeks to address the existing gap by systematically exploring the relationships between material properties and surface integrity across five different engineering materials. The selection was guided not only by their contrasting thermal conductivity and hardness values but also by their strong industrial relevance. Aluminum 6061 is widely used in aerospace and automotive components where lightweight design and smooth finishes are critical. Brass C26000 is used in electrical connectors and decorative parts, where surface consistency ensures both conductivity and aesthetics. Bronze C51000 is employed in bushings and bearings for mechanical systems that require stable tribological performance. Carbon Steel 1020, a structural and general engineering material, demands surface stability in load-bearing applications. Stainless Steel 304 is widely used in biomedical implants, food processing equipment, and corrosion-resistant structures, where surface integrity directly affects hygiene, fatigue life, and wear resistance. By delivering a thorough analysis of how thermal and mechanical properties influence machined surface quality, this research provides essential insights for refining machining processes to enhance component performance.

## Literature Review

Surface integrity in CNC machining has been widely studied due to its direct impact on component performance and reliability^13^. Several studies have examined how machining parameters influence surface roughness and surface waviness, particularly in precision manufacturing^14^. Bigerelle et al. (2025) highlighted the importance of surface texture parameters in assessing the functional properties of machined components, emphasizing that both micro-scale surface roughness and macro-scale surface waviness play critical roles in determining part performance^15^. Čep et al. further demonstrated that surface roughness is influenced by cutting speed, feed rate, and tool geometry, with improper parameter selection leading to increased surface defects^16^. Sen et al.. investigated surface roughness in wire electrical discharge machining (WEDM), revealing that different wire feed rates significantly affect surface finish quality^17^. Their research highlights how precise control over machining parameters can enhance surface integrity, especially in CNC processes.

Material properties, such as hardness and thermal conductivity, significantly influence surface roughness and waviness in machining operations^18^. Bashir et al. demonstrated that harder materials tend to exhibit greater surface roughness due to increased cutting resistance and tool wear^19^. In contrast, materials with high thermal conductivity, such as Aluminum, often produce smoother surfaces due to better heat dissipation^20^. Ponce et al. studied the effects of material composition on surface roughness in aerospace alloys, confirming that high-hardness materials experience greater cutting forces and increased tool wear, leading to surface defects^21^. Alsoufi et al. analyzed the effects of different machining technologies on material surface properties, comparing abrasive waterjet (AWJ) and laser beam (LB) cutting in carbon steel^12^. Their study found that AWJ resulted in smoother surfaces due to its non-contact machining nature, whereas LB cutting exhibited rougher textures due to thermal effects. These findings underscore the role of thermal and mechanical properties in machining processes. Research has also shown that thermal conductivity directly affects machining-induced temperature variations, which, in turn, impact surface quality^22^. Low thermal conductivity materials like stainless steel experience localized heat buildup, causing thermal expansion and deteriorating surface integrity^23^. Meanwhile, materials with higher thermal conductivity, such as Aluminum and Brass, facilitate efficient heat dissipation, maintaining stable cutting conditions and reducing tool wear^24^. This emphasizes the need for tailored machining strategies based on material properties.

Machining parameters, including feed rate, cutting speed, and depth of cut, play a fundamental role in surface roughness and surface waviness formation^25^. Experimental investigations have shown that increasing the feed rate leads to higher surface roughness, as larger uncut chip thickness results in more pronounced tool marks on the workpiece^26^. Meanwhile, higher cutting speeds can improve surface quality by reducing built-up edge formation and enhancing material shearing efficiency^27^. However, excessive cutting speeds can generate excessive heat, negatively affecting tool longevity and surface integrity^28^. Studies have also revealed that the depth of cut influences surface waviness, where deeper cuts increase the likelihood of surface irregularities due to higher cutting forces^29^.

Recent studies continue to advance the understanding of machining-induced surface roughness, particularly in turning operations under dry and semi-dry conditions. Kónya et al. investigated the effects of cutting speed and feed rate on surface roughness parameters during dry turning of austenitic stainless steel, showing that feed rate had a dominant influence on roughness and cutting force^30^. However, their analysis focused on a single material system and primarily reported mean roughness trends, without addressing cross-material variability or waviness behavior. Pasic et al. demonstrated that, even under controlled depths of cut and varied cutting parameters, surface roughness in turning exhibits strong sensitivity to machining conditions, especially when analyzed through predictive modeling and hybrid optimization methods^31^. While this work provides valuable insights into parametric sensitivity, it does not consider multi-material comparisons under identical cutting parameters nor examine statistical surface descriptors beyond average values. In studies on Inconel 718, Szablewski et al. provided a comparative assessment of how tool wear and cutting parameters influence surface topography and roughness, confirming the significant role of cutting variables in machining-induced surface quality in high-strength alloys^32^. Nevertheless, this investigation is limited to a single high-strength alloy and does not establish a common processing baseline across different material classes. These recent contributions provide an updated experimental context for machining-induced roughness formation and support the motivation for the present multi-material comparative study conducted under identical turning conditions.

Despite extensive research on machining-induced surface roughness, limited studies have examined the combined effects of material properties and machining parameters on surface roughness and waviness. Most prior work has focused on single-material investigations, making generalizing results across different alloys challenging. Furthermore, the influence of thermal conductivity on surface integrity remains underexplored, especially in comparative multi-material studies^33^. In addition, few studies quantitatively assess how material properties affect not only the magnitude of roughness, but also its variability, distribution shape, and functional character.

Although a recent kinematics-based and machine-learning framework predicted surface roughness in three materials during CNC turning, it remained material-specific. It did not address waviness or multi-material comparison^34^. In addition, the turning parameters for surface integrity using multi-criteria decision tools are optimized in^35^, but their study is limited to a single chromium–nickel alloy. Despite these advances, systematic, multi‑material comparative studies that jointly assess roughness, waviness, profile ratios, skewness, and kurtosis under unified CNC‑turning conditions remain absent. The present work directly addresses this gap by establishing an identical-parameter, multi-material turning dataset and interpreting the results using a metrology-informed framework that captures magnitude, variability, and statistical structure of surface integrity. This gap highlights the novelty and necessity of the present investigation.

## Methodology

### Experimental setup

**Fig. 1:**
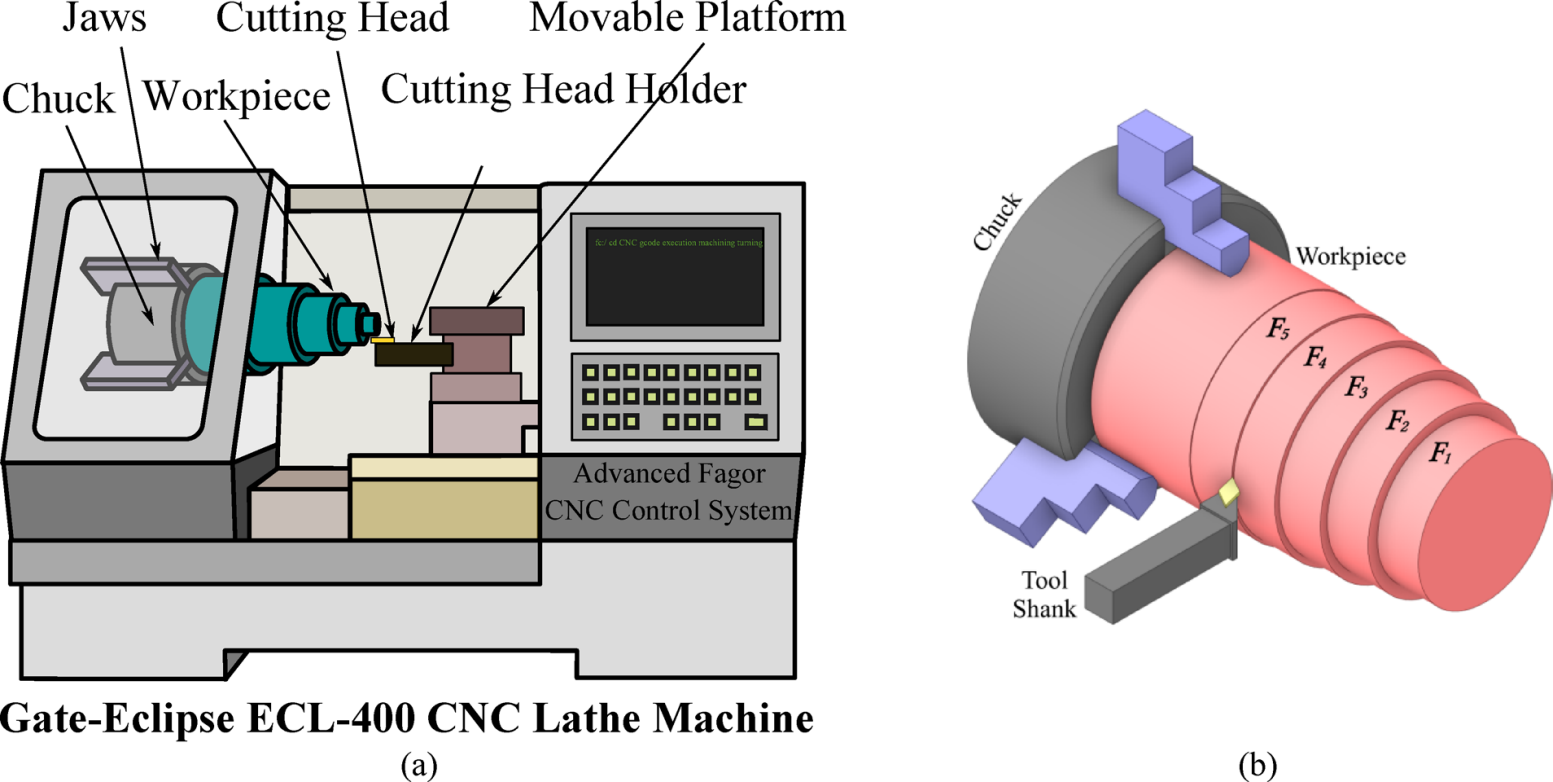
Schematic diagram of **(a)** machining setup and **(b)** workpiece closeup view.

The machining experiments were conducted using a high-precision Gate-Eclipse ECL-400 CNC lathe machine, a robust and rigid system designed to achieve high machining accuracy and repeatability. Equipped with an advanced Fagor CNC control system, the lathe machine provided precise adjustments to cutting speeds, feed rates, and depths of cut, ensuring controlled machining conditions across all trials. Figure 1 present a schematic diagram of the machining setup and workpiece positioning. Figure 1(a) illustrates the lathe CNC machine, cutting head and holder, chuck, workpiece and the direction of cutting. Figure 1(b) provides a close-up view of the workpiece and tool shank, highlighting the five axial zones *F*_1_-*F*_5_ used for measurements. Zones are ordered from the free end (*F*_1_) toward the chuck (*F*_5_), i.e., from furthest to nearest to the chuck, along the cutting direction.

To evaluate the influence of machining parameters on material removal rate (MRR) and surface integrity, a range of cutting speeds (30, 60, 90, and 120 m/min) and feed rates (0.05, 0.10, 0.15, and 0.20 mm/rev) were employed, while the depth of cut was maintained at a constant 0.25 mm for all experiments. This approach ensured consistency while enabling a thorough investigation of how varying speeds and feeds affected machining performance.

The cutting depth was set at 0.25 mm for all materials to remove geometric variations and focus on the effects of intrinsic material properties like hardness and thermal conductivity. This consistent parameter also facilitated uniform tool engagement and stable force conditions during dry machining, which is essential for a valid analysis of surface integrity. Likewise, the chosen cutting speed range (30–120 m/min) reflects typical values used in dry turning of engineering alloys, selected to prevent tool overheating or excessive wear while ensuring comparability among various materials. The choice to use the same cutting parameters for all materials, instead of customizing them, was a deliberate methodological strategy to emphasize the influence of intrinsic thermal and mechanical properties. It is recognized that this approach does not mirror best industrial practices, where machining would occur under optimized conditions for each material. Hence, this study aimed to establish a uniform processing environment, allowing differences in surface integrity to be attributed mainly to material characteristics instead of adjustments in process variables.

The multi-axis capability of the CNC system allowed for efficient and repeatable cutting operations, while the integrated cooling system maintained thermal stability, minimizing tool wear during prolonged machining. Workpieces were securely mounted in a high-rigidity chuck to prevent vibrational effects on surface quality, ensuring consistent and accurate machining outcomes. Each engineering material underwent turning under dry machining conditions to assess its inherent machinability accurately without the influence of external lubricants, ensuring that the observed thermal effects stemmed primarily from material properties. Environmental conditions were carefully controlled throughout the experiments, with the laboratory temperature maintained at 22 ± 1 °C and relative humidity at 45 ± 5%, eliminating external variables that could affect machining consistency and surface measurements.

Prior to the experimental runs, the CNC lathe underwent a comprehensive calibration procedure using a certified reference workpiece to verify spindle alignment, feed rate accuracy, and tool positioning. This ensured positional accuracy within ± 0.01 mm and minimized geometric deviations during machining. Additionally, tool inserts were inspected under a metallurgical microscope before each trial to prevent the influence of pre-existing wear or edge chipping on the results.

All machining trials used ISO-standard carbide inserts with TiAlN coatings (CNMG 120408). The tool geometry was as follows: rake angle − 6°, clearance (flank) angle 6°, helix angle 0°, and nose radius 0.8 mm. These values were kept constant across all tests to minimize variability and ensure that differences in surface integrity were attributable to material properties rather than tool geometry.

Tool wear was controlled through pre-trial microscopic inspection and limited insert usage (≤ 2 samples per insert). Quantitative flank wear (VB) measurements and SEM-based wear characterization were not performed in the present study, as machining was intentionally conducted within the initial wear regime to avoid progressive tool degradation. This strict limitation and inspection protocol ensured that the measured surface roughness values reflected intrinsic material behavior under controlled conditions rather than progressive tool degradation. While quantitative flank wear data were not recorded, the conservative replacement strategy provides confidence that roughness results are representative and comparable across materials. Inserts showing any flank wear, built-up edge, or micro-chipping were immediately replaced to avoid progressive degradation. Chip formation was monitored qualitatively during each trial to identify gross adhesion, built-up edge (BUE), or unstable segmentation. While chip images were not systematically documented in this study, qualitative observations guided the interpretation of surface integrity results, particularly for materials prone to adhesion (e.g., Aluminum 6061) or exhibiting stable strain hardening (e.g., Stainless Steel 304). Although real-time wear monitoring was not implemented, this replacement protocol and the chosen speed/feed ranges, selected to avoid overheating, maintained stable conditions across materials for a fair multi-material comparison. This design choice prioritized a uniform baseline across alloys, reserving quantitative wear tracking for a dedicated follow-up study.

Since the study focuses on quantitative surface characterization using profilometry, the primary assessment of surface condition is based on numerical roughness and waviness descriptors. Representative schematic illustrations of the turned sample geometry and machining configuration can be found in our previously validated work^36^, which helps illustrate the specimen geometry and machining configuration. All surface analyses in this study were based on profilometry and statistical descriptors. Although 3D areal surface parameters (ISO 25178) provide comprehensive spatial characterization, the present study focuses on ISO 4287 profile-based descriptors, which are well suited for the predominantly directional textures generated by turning; extension to areal parameters is reserved for future investigations.

### Material tested

The study investigated five engineering materials, each chosen for its unique combination of mechanical and thermal properties, covering a spectrum of machining behaviors from highly machinable, thermally conductive metals to harder, lower-conductivity alloys, as presented in Table 1. Aluminum alloy 6061, with its high thermal conductivity of 205 W/m·K and low hardness of 95 HBW, offers excellent machinability, low cutting resistance, and the potential for high-quality surface finishes, making it widely used in aerospace and automotive applications. Brass C26000, featuring a thermal conductivity of 109 W/m·K and a hardness of 115 HBW, exhibited moderate cutting resistance and high ductility, making it suitable for precision electrical components and decorative applications. Bronze C51000, with a lower thermal conductivity of 60 W/m·K and hardness of 112.5 HBW, presented slightly higher cutting resistance but remained a favorable choice for applications requiring wear resistance, such as bushings and bearings. Carbon steel 1020 annealed, with a thermal conductivity of 50 W/m·K and a hardness of 150 HBW, balanced strength, and machinability, making it applicable in structural and general mechanical uses, though it exhibited increased cutting resistance. Stainless steel 304 annealed, the hardest and least thermally conductive material in the study, with a hardness of 210 HBW and thermal conductivity of only 16 W/m·K, presented significant machining challenges due to its tendency for heat accumulation and rapid tool wear. The selection of these materials ensured a diverse evaluation of how different thermal and mechanical characteristics influence machining outcomes and surface integrity.

**Table 1:** Mechanical and thermal properties of materials chosen for the study.

Property	Aluminum Alloy 6061	Brass C26000	Bronze C51000	Stainless Steel 304 Annealed	Carbon Steel 1020 Annealed
Thermal conductivity (W/m·K)	205	109	60	16	50
Hardness (HBW)	95	115	112.5	210	150

The thermal conductivity values reported in Table 1 were not measured experimentally in this study. Instead, they were obtained from manufacturer/vendor datasheets and cross-verified with published literature ranges for each material. As such, no laboratory measurement steps or equipment were employed here.

### Surface integrity assessment

**Fig. 2:**
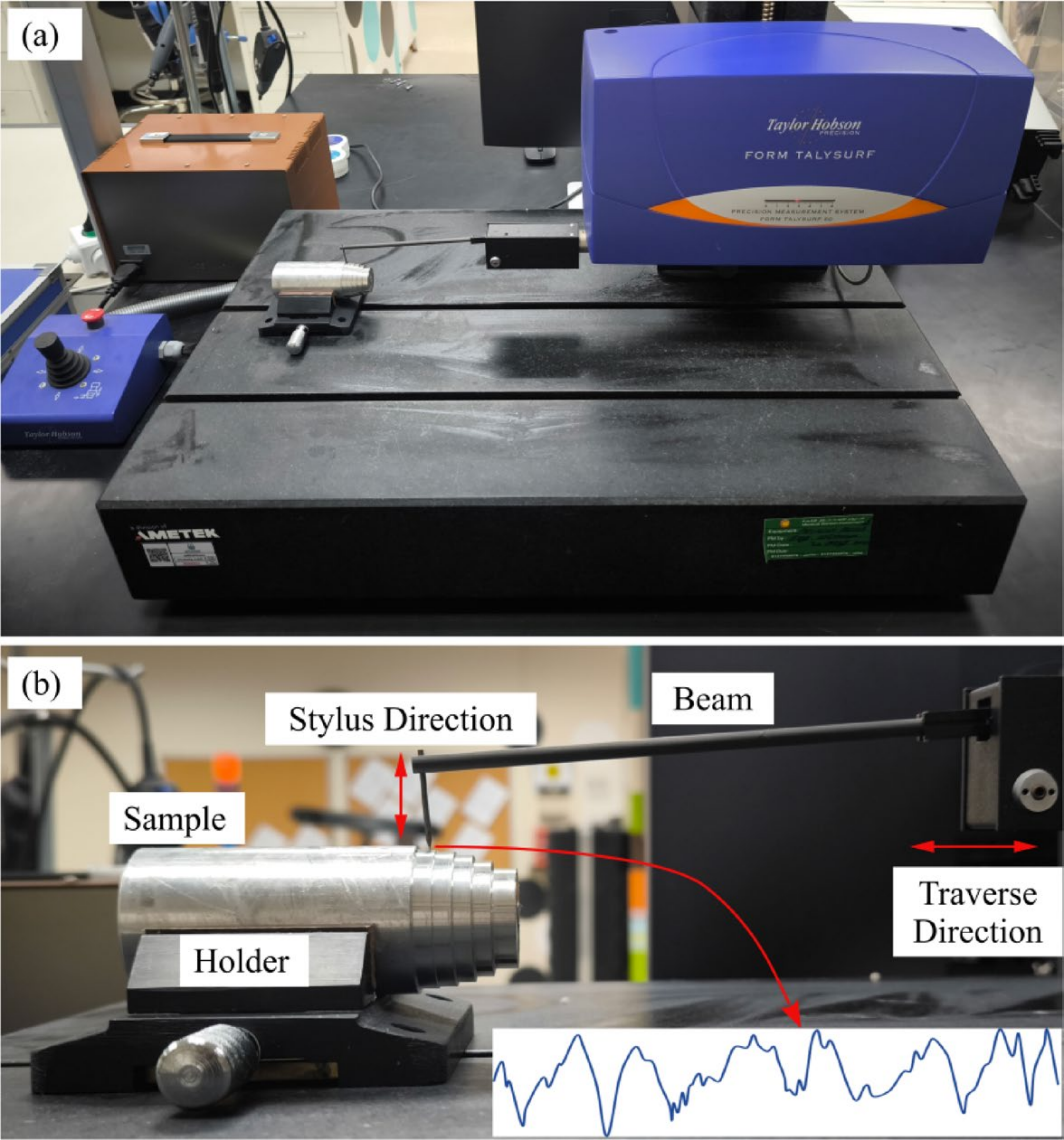
Surface roughness measurement using a Taylor Hobson Form Talysurf^®^ stylus profilometer. **(a)** Overall view of the instrument on the granite base. **(b)** Close-up of the cylindrical specimen mounted in the holder with the stylus poised on the surface and the traverse beam visible.

The surface integrity of the machined samples was evaluated using a high-precision Taly-Surf^®^ profilometer (Taylor Hobson Precision, Inc.), capable of capturing surface texture measurements at nanometer-level resolution. Figure 2 provides a photographic view of the profilometer and setup. Figure 2(a) shows the overall system on the granite base, and Fig. 2(b) shows the sample mounted in the holder with the stylus poised on the surface. The arrows indicate the stylus direction and the traverse direction. The inset in the figure displays a representative 2D line profile acquired in the same orientation. The profilometer stylus was aligned parallel to the axis of the cylindrical workpiece, following ISO 4287/4288 recommendations for turned surfaces. Measurements were taken at the mid-span of the machined section to avoid the entry and exit regions, where transient effects occur. This orientation captures the feed-induced lay pattern, whereas perpendicular scans could underrepresent the roughness. Each measurement was repeated at three different circumferential locations, and the average values were reported to ensure representativeness. This stylus-based instrument provided accurate readings of surface roughness (*R*_a_), waviness (*W*_a_), skewness (*R*_sk_, *W*_sk_), and kurtosis (*R*_ku_, *W*_ku_), enabling a comprehensive analysis of the surface features induced by the machining process. The profilometer was configured with a 2 μm conisphere stylus to ensure precise surface tracing, a measurement force of 0.7 mN to prevent surface deformation during scanning, and a traverse speed of 0.5 mm/s to maintain consistent measurement conditions across all samples. Although 2D roughness traces were recorded for all specimens, the present study reports only the statistical descriptors (*R*_a_, *W*_a_, *R*_sk_, *R*_ku_, and their ratios), which provide standardized and reproducible comparisons across materials. Representative 2D profiles can be provided in supplementary form in future work to complement the statistical outcomes visually.

Three independent measurements were taken at three distinct locations for each sample to capture its inherent surface variability. The averaged values minimized localized anomalies and enhanced the reliability of the data. Data processing included applying linear least-squares regression to eliminate baseline drift. Furthermore, a Gaussian low-pass filter is used in accordance with ISO 4287 standards to effectively separate micro-scale surface roughness from macro-scale surface waviness components. This ensured that the analysis accounted for both short-wavelength tool marks and longer-wavelength undulations, providing a comprehensive evaluation of surface integrity.

Prior to measurements, the profilometer was calibrated using a certified roughness standard (*R*_a_ = 1.00 ± 0.02 μm), ensuring traceability to national metrology standards and maintaining measurement accuracy. Calibration checks were repeated after every ten measurements to prevent instrument drift over extended use.

An uncertainty analysis was conducted in accordance with the ISO Guide to the Expression of Uncertainty in Measurement (GUM) to ensure confidence in the measurement results. The combined standard uncertainty ($\:{U}_{c}$) was calculated by considering the profilometer’s instrument precision, measurement repeatability, and calibration standard deviation:

$$\:{U}_{c}=\sqrt{{U}_{instrument}^{2}+{U}_{repeatability}^{2}+{U}_{calibration}^{2}}$$

Where:

#### $\:{U}_{instrument}$

0.02 μm (based on manufacturer specifications).

#### $\:{U}_{repeatability}$

0.015 μm (from repeated measurements).

#### $\:{U}_{calibration}$

0.01 μm (from the calibration certificate).

This results in *U*_c_ = 0.027, which will be used as a baseline and compared to the uncertainty obtained from experimental results. This combined uncertainty represents the calibration and instrument-related uncertainty of the profilometer rather than the experimental scatter of repeated roughness measurements and is at least one order of magnitude smaller than the measured roughness values. Therefore, its contribution to total variance is negligible and does not affect the statistical confidence or repeatability of the reported surface roughness and waviness results.

## Results and discussion

The collected data were analyzed using OriginPro 2024, a statistical software suite that enabled detailed regression modeling and trend analysis. Descriptive statistics, including mean values, standard deviations, and variance calculations, were applied to evaluate the consistency of surface roughness and surface waviness metrics across various machining conditions. Multivariate regression models were developed to examine the correlation among machining parameters, material properties, and surface integrity characteristics. These models incorporated thermal conductivity, hardness, feed rate, cutting speed, and depth of cut as key independent variables influencing surface roughness and waviness outcomes. Residual analysis was performed to validate the predictive accuracy of the regression models, particularly for harder materials such as stainless steel 304 annealed, where deviations were anticipated due to heat accumulation and increased cutting resistance. Scatter plots and trend analyses were utilized to visualize relationships between machining parameters and surface features, ensuring clear interpretations of underlying machining behaviors. Additionally, 2D topographical surface texture maps were generated to visually represent roughness distributions across different materials, highlighting variations in texture uniformity.

### Surface roughness and waviness across materials

**Fig. 3:**
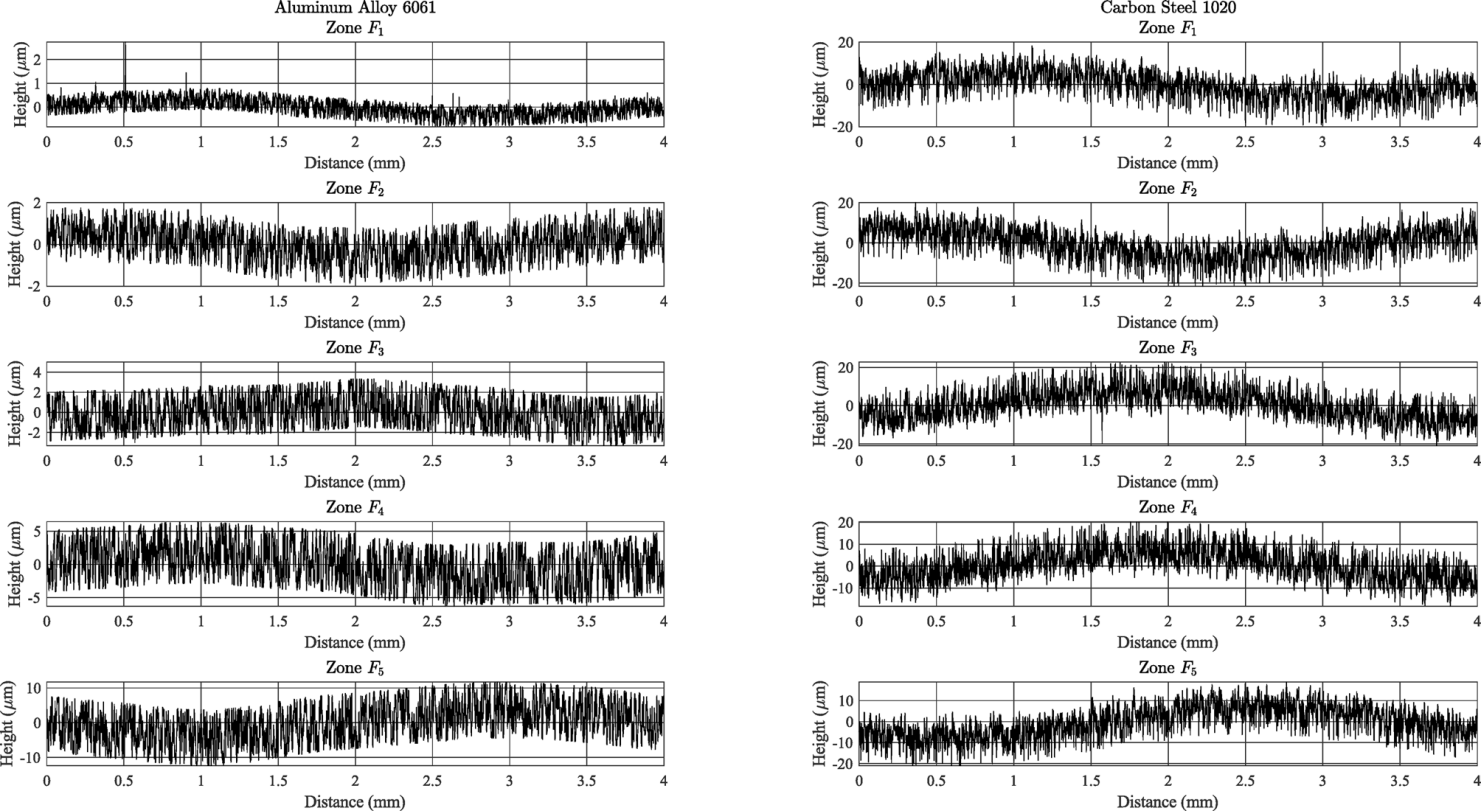
Representative 2D line profiles parallel to the turning axis for zones *F*_1_–*F*_5_. Left column is for Aluminum Alloy 6061, while right column is for Carbon Steel 1020.

To provide visual context for the standardized statistics, Fig. 3 shows representative 2D line profiles collected parallel to the turning axis for two materials that span our roughness range (Aluminum Alloy 6061 and Carbon Steel 1020) across zones *F*_1_–*F*_5_. All traces use the same ISO 4287/4288 filtering and evaluation settings as in § 3.3, enabling direct visual comparison. The qualitative features visible in these traces are consistent with the quantitative parameters reported below.

**Fig. 4:**
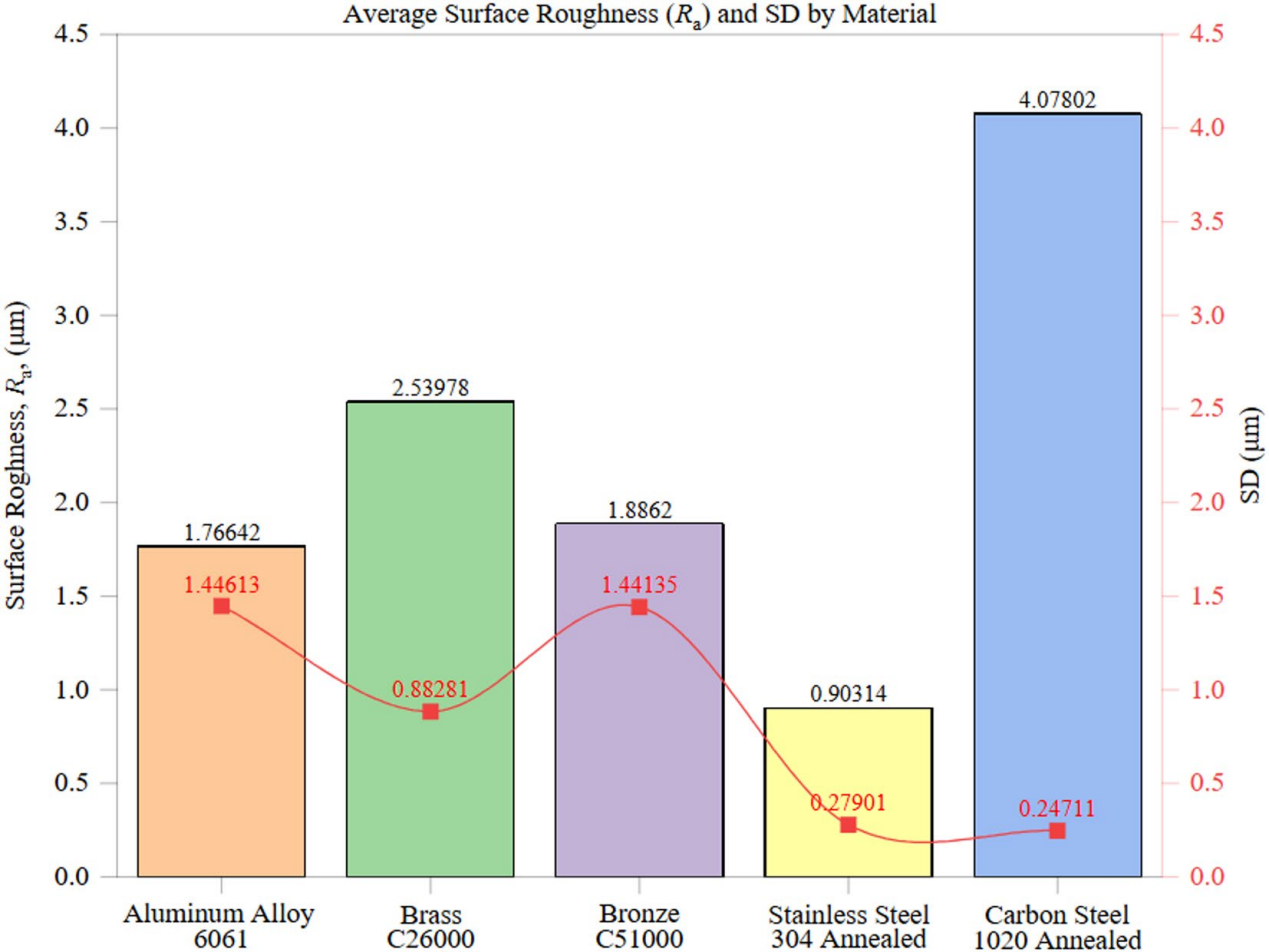
Average Surface Roughness (*R*_a_) and SD by Material.

Figure 4 presents the average arithmetic surface roughness (*R*_a_) and corresponding standard deviation (SD) for each tested material, highlighting significant variations in surface finish under identical machining conditions. These variations are governed by the complex interplay between material hardness, thermal conductivity, chip formation mechanisms, and tool-material interactions.

The standard deviation values across materials offer key insights into machining consistency. While Stainless Steel 304 maintained a low *R*_a_ and stable finish, Bronze C51000 and Aluminum 6061 experienced pronounced fluctuations due to tool wear progression, chip formation instability, and thermal expansion effects during extended machining cycles. Notably, Carbon Steel 1020 had both the highest *R*_a_ and one of the lowest CVs, indicating consistently rough surfaces rather than random variations, a critical consideration in process planning.

Among the tested materials, Stainless Steel 304 exhibited the lowest *R*_a_ at 0.9031 ± 0.2790 μm, with a coefficient of variation (CV) of 30.9%, indicating a consistent surface finish across the machined zones. This stability is attributed to the material’s moderate hardness (210 HBW) and low thermal conductivity (16 W/m·K), which promote uniform heat dissipation, reducing built-up edge (BUE) formation. Furthermore, the strain-hardening capability and fine-grained microstructure of Stainless Steel 304 enhance cutting stability by mitigating vibrations and tool chatter. Compared to Carbon Steel 1020, Stainless Steel 304 achieved a 77.9% lower *R*_a_, underscoring its superior machinability. This performance was further supported by chip observations, where Stainless Steel 304 produced uniform, curled chips with minimal adhesion, which is consistent with its strain-hardening capacity and stable surface finish.

In contrast, Carbon Steel 1020 demonstrated the highest *R*_a_ of 4.0780 ± 0.2471 μm (CV: 6.06%), reflecting a consistently rough surface finish with minimal variability. This elevated roughness is primarily due to the steel’s moderate hardness (150 HBW) and thermal conductivity (50 W/m·K), which increase cutting forces and friction. Moderate thermal conductivity promotes localized heating, accelerates tool wear, and leads to surface irregularities. The high Ra indicates that tool degradation and chip adhesion are prevalent during machining, which severely impacts surface integrity. Qualitative chip monitoring confirmed this behavior, as Carbon Steel 1020 generated unstable, continuous chips with evident adhesion and oxidation traces, reinforcing the link between chip morphology and consistently rough surfaces.

Aluminum 6061 exhibited an average *R*_a_ of 1.7664 ± 1.4461 μm (CV: 81.9%), indicating substantial variability across different measurement zones. Despite its high thermal conductivity (205 W/m·K), facilitating efficient heat dissipation, Aluminum’s softness (95 HBW) and tendency to form BUE and micro-burrs during cutting contribute to significant surface roughness fluctuations. These findings highlight that while high thermal conductivity typically enhances the surface finish, Aluminum’s ductility and adhesion propensity can offset these benefits. Compared to Brass C26000, Aluminum 6061 achieved a 30.5% lower average *R*_a_ but had a 46.6% higher CV, emphasizing the trade-off between average roughness and consistency. The variability observed in Aluminum Alloy 6061 (CV ≈ 82%) is consistent with chip-level observations, which reveal built-up edge (BUE) and adhered fragments, helping to explain the roughness fluctuations despite efficient heat dissipation.

Brass C26000 recorded a moderate *R*_a_ of 2.5398 ± 0.9870 μm (CV: 38.9%). Its moderate hardness (115 HBW) and high thermal conductivity (109 W/m·K) aid in heat dissipation and reduce tool wear, resulting in relatively consistent finishes. However, chip adhesion and the ductile nature of Brass can introduce minor surface irregularities, accounting for the moderate variability observed. In comparison, Bronze C51000 exhibited a 25.8% lower *R*_a_ of 1.8862 ± 1.6115 μm (CV: 85.4%), though with significantly higher variability. Bronze’s higher hardness (115 HBW) mitigates adhesion but leads to occasional tool vibrations, contributing to the substantial roughness variation.

Comparisons with prior literature (e.g., Petropoulos et al.^12^; Krolczyk et al.^14^ validate these findings. Materials with thermal conductivity above 100 W/m·K (such as Aluminum and Brass) generally yielded smoother finishes but exhibited inconsistencies due to adhesion-related phenomena. Conversely, materials with higher hardness (≥ 150 HBW) consistently produced rougher surfaces (*R*_a_ ≥ 4.0 μm), aligning with the observations of Özel and Karpat^15^ on the impact of increased cutting resistance on surface integrity.

From an industrial perspective, these results underscore the importance of material-specific machining strategies. For components requiring high dimensional accuracy and minimal surface roughness, Stainless Steel 304 proves advantageous. Meanwhile, materials like Carbon Steel 1020 and Brass C26000 necessitate optimized cutting speeds, feed rates, and advanced tool coatings to mitigate roughness-related issues and enhance overall process efficiency.

**Fig. 5:**
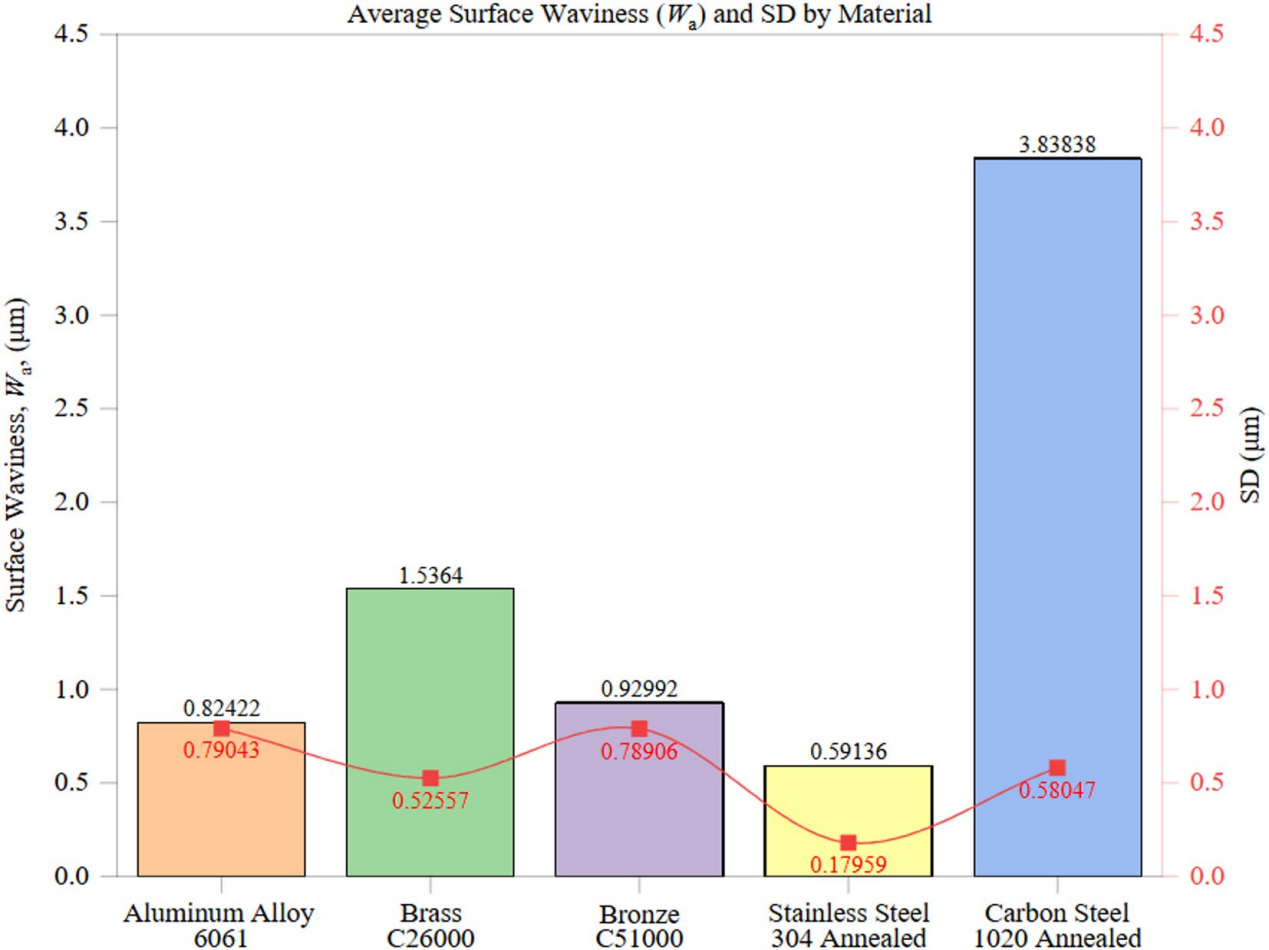
Average Surface Waviness (*W*_a_) by Material.

Figure 5 illustrates the average surface waviness (*W*_a_) and corresponding standard deviation (SD) for each tested material, providing insights into how material properties and machining conditions affect large-scale surface irregularities. The waviness trends reflect the combined influence of thermal conductivity, hardness, tool-material interactions, and process stability.

The standard deviation values highlight machining consistency across materials. Stainless Steel 304’s low SD and CV indicate stable cutting conditions, making it favorable for applications requiring consistent surface integrity. In contrast, Carbon Steel 1020, while having a high average *W*_a_, demonstrated a relatively moderate CV, indicating that rough surfaces were consistently produced rather than randomly occurring. Aluminum 6061 and Bronze C51000, despite their moderate average *W*_a_ values, showed high variability, reflecting the significant influence of chip formation dynamics, adhesion effects, and tool wear progression.

Among the examined materials, Stainless Steel 304 exhibited the lowest *W*_a_, averaging 0.5914 ± 0.2008 μm with a coefficient of variation (CV) of 33.9%, indicating a highly uniform waviness profile. Despite its low thermal conductivity (16 W/m·K) and high hardness (210 HBW), the material’s strain-hardening ability and stable cutting behavior under controlled conditions helped suppress excessive waviness. While the low thermal conductivity might lead to localized heat accumulation, the material’s resistance to adhesion and consistent tool engagement contributed to maintaining a smooth waviness profile. This observation was consistent with chip morphology, where Stainless Steel 304 produced uniform, curled chips with stable flow, reducing the risk of periodic tool–chip instabilities that typically manifest as waviness.

In contrast, Carbon Steel 1020 demonstrated the highest *W*_a_, with an average of 3.8384 ± 0.5805 μm (CV: 15.1%), highlighting pronounced waviness across different machining zones. The interplay between its moderate thermal conductivity (50 W/m·K), relatively high hardness (150 HBW), and ferritic-pearlitic microstructure causes irregular chip formation and fluctuating tool engagement. These factors lead to periodic waviness patterns, exacerbated by accelerated tool wear and increased cutting force variations, particularly at higher feed rates and cutting speeds. Qualitative chip inspection revealed unstable continuous chips with adhesion layers in Carbon Steel 1020, which likely contributed to periodic force fluctuations and the pronounced waviness patterns observed.

Brass C26000 displayed a moderate *W*_a_ of 1.5364 ± 0.5256 μm (CV: 34.2%), which is attributed to its high thermal conductivity (109 W/m·K) and moderate hardness (~ 100 HBW), which promotes efficient heat dissipation and stable cutting conditions. Meanwhile, its ductile nature makes it susceptible to chip adhesion and deformation at the tool-material interface, slightly increasing surface undulations.

Bronze C51000 exhibited a *W*_a_ of 0.9299 ± 0.7891 μm (CV: 84.9%), suggesting significant variability in waviness despite having a lower average than Brass. This can be attributed to Bronze’s higher hardness (115 HBW), which mitigates adhesion-related effects but increases the risk of vibration-induced waviness. Occasional tool engagement inconsistencies at higher feed rates may explain the observed fluctuations.

Aluminum Alloy 6061, known for its excellent machinability and high thermal conductivity (205 W/m·K), recorded a *W*_a_ of 0.8242 ± 0.79043 μm (CV: 95.9%). Despite its superior heat dissipation, Aluminum’s low hardness (95 HBW) and the tendency for built-up edge (BUE) formation significantly affected waviness consistency. The presence of micro-burrs and elastic deformation under cutting loads further contributed to periodic waviness, especially at higher feed rates. This variability underscores the challenge of balancing high conductivity with material softness. The high variability in Aluminum Alloy 6061 waviness (CV ≈ 96%) was consistent with chip morphology marked by BUE and adhesion, which periodically disrupted tool–chip contact and generated long-wavelength undulations.

Comparative analysis with prior literature confirms that materials with higher thermal conductivity (> 100 W/m·K) generally yield smoother waviness profiles, although exceptions like Aluminum 6061 arise due to BUE formation and ductility. On the other hand, harder materials (≥ 150 HBW), like Carbon Steel 1020, tend to exhibit more pronounced waviness due to increased cutting resistance and higher tool wear rates, consistent with findings from Alsoufi et al.^12^ and Alyas, R. R^14^. It should be noted that Brass C26000’s increased waviness compared to Bronze C51000 highlights the compromise between ductility-induced deformation and hardness-induced vibrations.

From an industrial standpoint, these results highlight the importance of optimizing cutting parameters to control waviness. Materials like Stainless Steel 304 offer stable surface profiles suitable for high-precision applications, whereas Aluminum 6061 and Bronze C51000 require adjusted feed rates and tool coatings to minimize waviness variability. Implementing advanced cutting strategies and vibration-dampening measures can substantially improve surface integrity for materials like Carbon Steel 1020, where waviness significantly affects part performance.

**Fig. 6:**
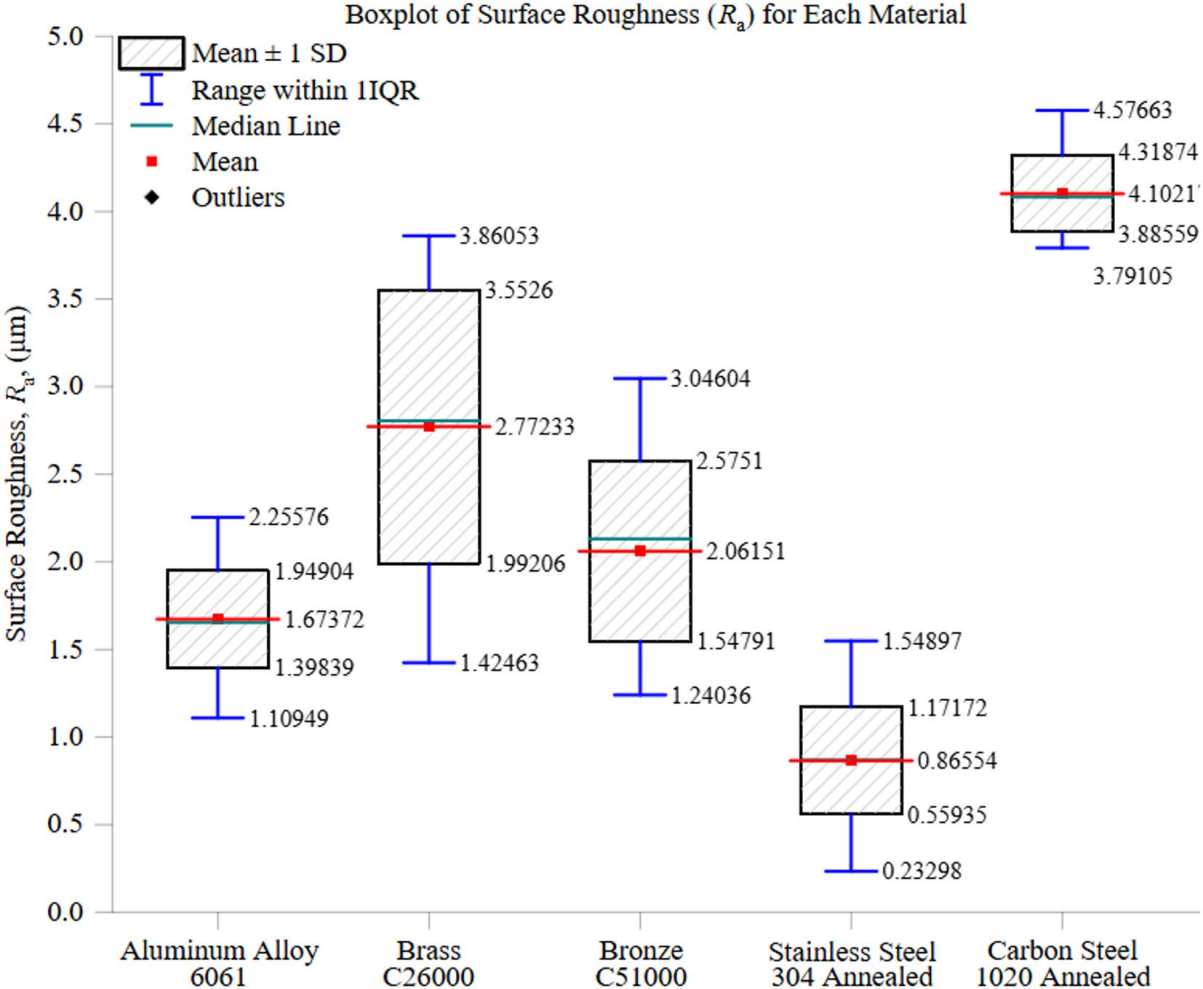
Boxplot of surface roughness (*R*_a_) for each material under identical machining conditions.

Figure 6 shows the statistical distribution of surface roughness (*R*_a_) across five engineering materials. It includes central tendency metrics (mean and median), the spread measured by the interquartile range (IQR), and variability bands represented by mean ± 1 standard deviation. When analyzed with thermal and mechanical properties, these statistical indicators offer valuable insights into each material’s surface integrity during CNC turning.

Carbon Steel 1020 exhibits the highest surface roughness, with a mean *R*_a_ around 4.1 μm and a slightly lower median, indicating a positively skewed distribution. The narrow IQR and the ± 1 SD range (3.9–4.3 μm) demonstrate minimal variability across trials. This consistency likely stems from uniform chip formation under moderate hardness (approximately 140 HBW) and low thermal conductivity (~ 50 W/m·K). Nevertheless, the heat retention in the cutting zone speeds up tool wear and increases roughness levels.

Stainless Steel 304 shows the lowest average *R*_a_ at approximately 0.9 μm, with a close correspondence between mean and median values and a narrow interquartile range (IQR), indicating strong consistency. The ± 1 standard deviation (SD) range is also tight, between 0.6 and 1.2 μm, which underscores its stable machining behavior. Although it has a very low thermal conductivity of about 16 W/m·K, the material benefits from a commendable strain-hardening rate, an austenitic structure, and a high hardness of around 200 HBW. These properties help prevent built-up edge (BUE) formation and facilitate clean shearing, resulting in a superior finish.

Aluminum 6061 features the second lowest mean *R*_a_ of approximately 1.7 μm, and its median closely aligns with the mean, indicating a high degree of symmetry. The interquartile range (IQR) and ± 1 standard deviation range (1.4–1.9 μm) reflect low variability. While its high thermal conductivity (~ 205 W/m·K) facilitates quick heat dissipation, its relatively low hardness (~ 95 HBW) renders it susceptible to built-up edge (BUE) and smearing effects, particularly during aggressive cutting, leading to inconsistent roughness across trials.

Bronze C51000 has an average *R*_a_ of approximately 2.1 μm, with a slightly higher median. A wider interquartile range (IQR) and ± 1 standard deviation (SD) band (1.5–2.6 μm) are observed, suggesting increased variability. This variability may result from its intricate microstructure, impacting chip segmentation and contact stability. With intermediate hardness (~ 110 HBW) and thermal conductivity (~ 60 W/m·K), it falls between Aluminum and carbon steel in terms of surface performance, although there are occasional variations in chip flow or adhesion.

Brass C26000 exhibits the greatest range and highest variability, with a mean *R*_a_ exceeding 2.8 μm, a widely varying IQR, and a ± 1 SD range of approximately 2 to 3.6 μm. This material is characterized by its ductility and relatively low hardness (around 100–110 HBW), which heightens its vulnerability to elastic recovery, tool vibration, and edge rounding during machining. Although it possesses high thermal conductivity (about 110 W/m·K), the alloy’s soft nature leads to inconsistent chip formation and an increased frequency of tool–material engagement issues.

The alignment of mean and median values and narrow IQRs in materials such as Stainless Steel 304 implies consistent and repeatable surface performance, making it suitable for precision manufacturing. The larger standard deviation bands and broader IQRs, particularly at Brass and Bronze, indicate that the challenge of machinability is not solely due to thermal factors but also due to microstructural behavior and mechanical compliance.

This analysis confirms that achieving an optimal surface finish relies on a synergistic balance between thermal conductivity, crucial for heat dissipation, and mechanical resistance, which affects chip shear and tool interaction. Materials that perform well in one aspect but poorly in the other may still exhibit subpar surface integrity without proper parameter adjustments.

While this study intentionally employed fixed machining parameters across all materials to isolate the effects of thermal conductivity and hardness on surface integrity, it is crucial to recognize that this approach deviates from best industrial practices, which typically involve parameter optimization for each material. As a result, the surface roughness and waviness values obtained here, particularly for materials like Carbon Steel 1020 and Brass C26000, tend to be higher than those reported in the literature under optimized conditions. For instance, prior studies indicate that Aluminum 6061 can achieve Ra values as low as 0.6–1.2 μm when machined at lower feed rates or with lubrication, compared to 1.77 μm observed here^14,25^. Similarly, Carbon Steel 1020 has been reported with *Ra* values between 2.5 and 3.5 μm under controlled environments, while our data reflect a consistent but rougher surface at ~ 4.08 μm^13^. These deviations underscore that the roughness differences observed in this study stem from intrinsic material properties rather than process parameter tuning.

Furthermore, while Stainless Steel 304 exhibited the lowest surface roughness and waviness values in this experiment, this outcome should not be misinterpreted as an indication of superior overall machinability. In practice, Stainless Steel is known for its high cutting resistance and poor thermal conductivity, which can lead to rapid tool wear, increased heat generation, and reduced tool life. The favorable surface integrity results observed here are likely due to the strain-hardening capability and stable chip formation of SS304 under the specific dry turning conditions used. Thus, surface finish alone does not comprehensively reflect machinability, which also depends on tool wear rates, cutting forces, chip morphology, and economic considerations.

These observations highlight the need for a balanced evaluation that considers both surface quality and process efficiency. While fixed-condition studies like this one are essential for establishing baseline material behavior, future research should include optimized cutting strategies and cost-performance analysis to characterize machinability across different material classes fully.

Theoretical surface roughness was also estimated using the classical kinematic–geometric model as a function of feed (*f*), *R*_a, theoretical_ = *f*^2^/(32*r*). For the present cutting conditions (*r* = 0.8 mm, *f* = 0.05–0.20 mm/rev), the theoretical *R*_a_ range is 0.1–1.6 μm. This corresponds well to the lowest experimental *R*_a_ values measured for Stainless Steel 304 (≈ 0.9 μm), but is considerably lower than the roughness values obtained for Carbon Steel 1020 and Aluminum Alloy 6061. These deviations demonstrate that, while the kinematic model provides an ideal baseline for the achievable finish, actual results are strongly influenced by material-dependent factors, such as adhesion, chip instability, and strain hardening.

To statistically validate the observed differences in surface roughness (*R*_a_) across the five materials, a one-way ANOVA was performed (Table 2). The analysis reveals a highly significant effect of material type on *R*_a_, with *p*-values less than or approximately equal to 0.05, indicating statistically significant differences at the 95% confidence level. This confirms that the variations in surface roughness are unlikely to arise from random experimental scatter and are instead driven by differences in intrinsic material properties under identical machining conditions. Moreover, the large effect sizes (*η*^2^ = 0.85; *ω*^2^ = 0.845) demonstrate that the statistically significant *p*-values are accompanied by substantial practical significance, with material type accounting for over 84% of the total variance in *R*_a_.

**Table 2:** One-way ANOVA results for *R*_a_ across the five materials.

Source	DF	Sum of Squares	Mean Square	F-value	*p*-value
Between Groups	4	179.33	44.832	205.83	1.02 × 10⁻^58^
Within Groups	145	031.58	00.218	–	–
Total	149	210.91	–	–	–

Subsequent Tukey HSD post-hoc comparisons (Table 3) further clarified the pairwise differences. Stainless Steel 304 produced significantly smoother surfaces than all other materials, particularly when compared with Carbon Steel 1020 (mean difference = − 3.57 μm, *p* < 0.001) and Aluminum Alloy 6061 (–1.14 μm, *p* < 0.001). These results confirm the exceptional stability and uniformity of Stainless Steel 304, despite its low thermal conductivity, as previously attributed to its strain-hardening capacity and stable chip formation.

Carbon Steel 1020 and Aluminum Alloy 6061, in contrast, showed significantly higher *R*_a_ values than the other materials. Carbon Steel 1020 was significantly rougher than Stainless Steel 304, Brass C26000, and Bronze C51000, with pairwise mean differences ranging from − 3.57 to + 1.00 μm (*p* < 0.001). This aligns with its combination of limited thermal conductivity and higher hardness, which promotes unstable chip flow and increased tool–workpiece interaction.

Aluminum Alloy 6061 produced significantly higher *R*_a_ values than Brass C26000 (mean difference = − 1.43 μm, *p* < 0.001) and Bronze C51000 (mean difference = − 0.72 μm, *p* = 0.011). The difference between Aluminum Alloy 6061 and Bronze C51000 was smaller in magnitude but still statistically significant (*p* = 0.011 < 0.05), confirming its adhesion-driven surface degradation and high variability. Among the copper-based alloys, Brass C26000 and Bronze C51000 were also statistically distinguishable (mean difference = + 0.38 μm, *p* < 0.001), although the difference was considerably smaller than for the other pairings. This reflects their broadly similar balance of hardness and conductivity, with Bronze achieving slightly smoother profiles overall.

**Table 3:** Tukey HSD post-hoc test results for *R*_a_ pairwise comparisons.

Comparison	Mean Difference (µm)	95% CI Lower	95%CI Upper	Adjusted *p*	Significant
Stainless Steel 304 vs. Carbon Steel 1020	–3.57	–3.24	–2.91	< 0.001	Yes
Stainless Steel 304 vs. Aluminum Alloy 6061	–1.14	–0.81	–0.48	< 0.001	Yes
Stainless Steel 304 vs. Brass C26000	–2.24	–1.91	–1.58	< 0.001	Yes
Stainless Steel 304 vs. Bronze C51000	–1.52	–1.20	–0.87	< 0.001	Yes
Carbon Steel 1020 vs. Aluminum Alloy 6061	+ 2.10	+ 2.43	+ 2.76	< 0.001	Yes
Carbon Steel 1020 vs. Brass C26000	+ 1.00	+ 1.33	+ 1.66	< 0.001	Yes
Carbon Steel 1020 vs. Bronze C51000	+ 1.71	+ 2.04	+ 2.37	< 0.001	Yes
Aluminum Alloy 6061 vs. Brass C26000	–1.43	–1.10	–0.77	< 0.001	Yes
Aluminum Alloy 6061 vs. Bronze C51000	–0.72	–0.39	–0.06	0.011	Yes
Brass C26000 vs. Bronze C51000	+ 0.38	+ 0.71	+ 1.04	< 0.001	Yes

Overall, the ANOVA and Tukey results reinforce the descriptive trends: Stainless Steel 304 consistently provided the smoothest and most uniform surfaces, Carbon Steel 1020 and Aluminum Alloy 6061 produced the roughest, while Brass C26000 and Bronze C51000 occupied intermediate positions. These findings statistically confirm that the interplay of thermal conductivity and hardness drives significant material-dependent differences in surface finish.

With this foundation established, the following section examines the correlations, interactions, and sensitivities that link material properties to these surface metrics.

### Influence of thermal and mechanical properties on surface integrity: correlation, interaction, and sensitivity analysis

**Fig. 7:**
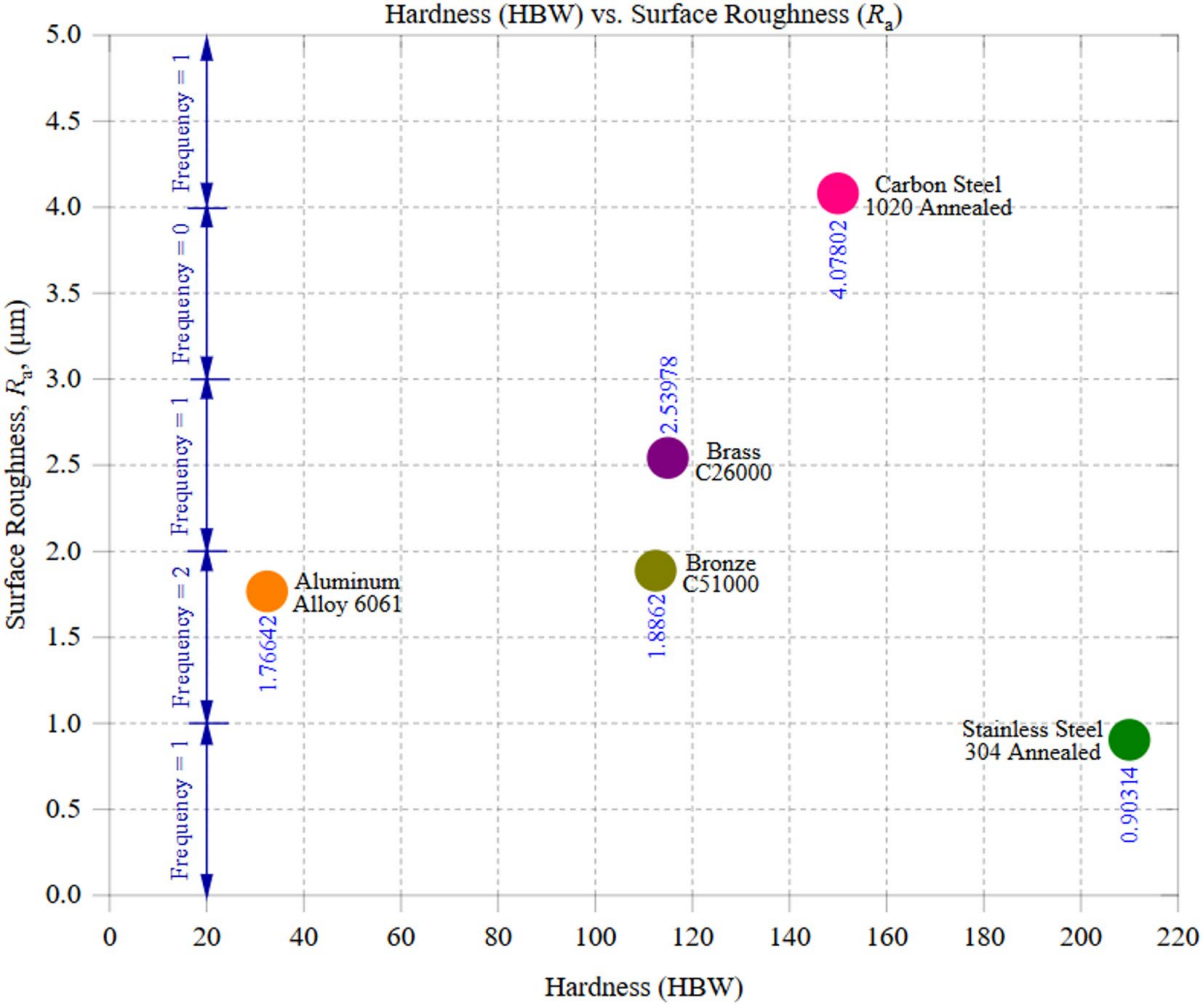
Hardness (HBW) vs. Surface Roughness (*R*_a_).

Figure 7 illustrates the correlation between material hardness (HBW) and the machined samples’ average surface roughness (*R*_a_), emphasizing the influence of mechanical properties on surface integrity. The data reveals an intricate relationship where material hardness does not always directly correlate with roughness, indicating the interplay of other factors such as thermal conductivity, tool wear, and machining dynamics. Among the tested materials, stainless steel 304, with the highest hardness of 210 HBW, exhibited the lowest surface roughness, averaging 0.90 μm with a standard deviation of 0.279 μm. Typically, harder materials tend to produce rougher surfaces due to increased cutting forces and tool wear; however, stainless steel’s behavior deviates from this expectation. Although low thermal conductivity would normally promote heat accumulation and deteriorate surface finish, Stainless Steel 304 exhibits pronounced strain-hardening behavior that promotes stable plastic flow and continuous curled chip formation, thereby suppressing built-up edge formation and surface tearing. The combination of low thermal conductivity (16 W/m·K) and high strain-hardening capacity likely contributed to stable material removal, minimizing cutting-edge damage and leading to a more uniform surface. Furthermore, the work-hardening effect of stainless steel enhances its resistance to deformation, reducing chatter and ensuring a more refined finish. Despite its high hardness, the low *R*_a_ suggests that machining conditions were well optimized, preventing excessive surface irregularities. In contrast, carbon steel 1020, with a hardness of 150 HBW, exhibited the highest surface roughness, averaging 4.08 μm with a standard deviation of 0.247 μm. The moderate thermal conductivity (50 W/m·K) combined with its ferritic-pearlitic microstructure contributed to increased tool wear and thermal expansion, which amplified the surface irregularities. Additionally, the combination of high friction and fluctuating cutting forces led to periodic roughness patterns, as observed in the machining zones. Intermediate materials, such as brass C26000 (115 HBW, *R*_a_ ≈ 2.54 μm) and Bronze C51000 (112.5 HBW, *R*_a_ ≈ 1.89 μm), exhibited moderate surface roughness values. Despite its higher thermal conductivity (109 W/m·K), Brass presented a slightly higher Ra than Bronze (60 W/m·K). This could be attributed to Brass’s higher ductility, which increases the likelihood of micro-tearing and chip adhesion during cutting, leading to localized surface irregularities. In contrast, Bronze’s combination of moderate hardness and lower conductivity likely resulted in steadier material removal, reducing its surface roughness. Aluminum alloy 6061, which has the lowest hardness (95 HBW) among the tested materials, exhibited a *R*_a_ of 1.77 μm, with a standard deviation of 1.45 μm. Although Aluminum 6061 exhibits high thermal conductivity (205 W/m·K), the observed roughness is higher than expected. This deviation stems from secondary effects such as built-up edge (BUE) formation and adhesion, which override the benefits of heat dissipation. The observed trends reinforce that hardness alone does not dictate surface roughness; instead, a combination of thermal properties, chip formation behavior, tool wear resistance, and machining parameters determines the final finish. For instance, harder materials like stainless steel can achieve lower roughness when thermal effects and machining conditions are well controlled. Conversely, softer materials like Aluminum may exhibit higher roughness due to adhesion effects and elastic deformation. These interpretations are consistent with chip-level observations: Aluminum Alloy 6061 exhibited a built-up edge and adhesion on chips, supporting its higher *R*_a_ variability, while Stainless Steel 304 produced stable, curled chips with minimal adhesion, reinforcing the explanation for its unusually low *R*_a_ despite its high hardness. The standard deviation values further support these findings, where materials with lower thermal conductivity (such as carbon steel) exhibit higher variability, although materials with superior thermal dissipation (such as Brass and Bronze) maintain more stable roughness values.

These results emphasize the need for optimized machining strategies tailored to each material’s physical and mechanical properties. Controlling feed rate, tool geometry, and cutting speed is essential to achieving the desired surface roughness, ensuring improved performance and longevity of machined components.

**Fig. 8:**
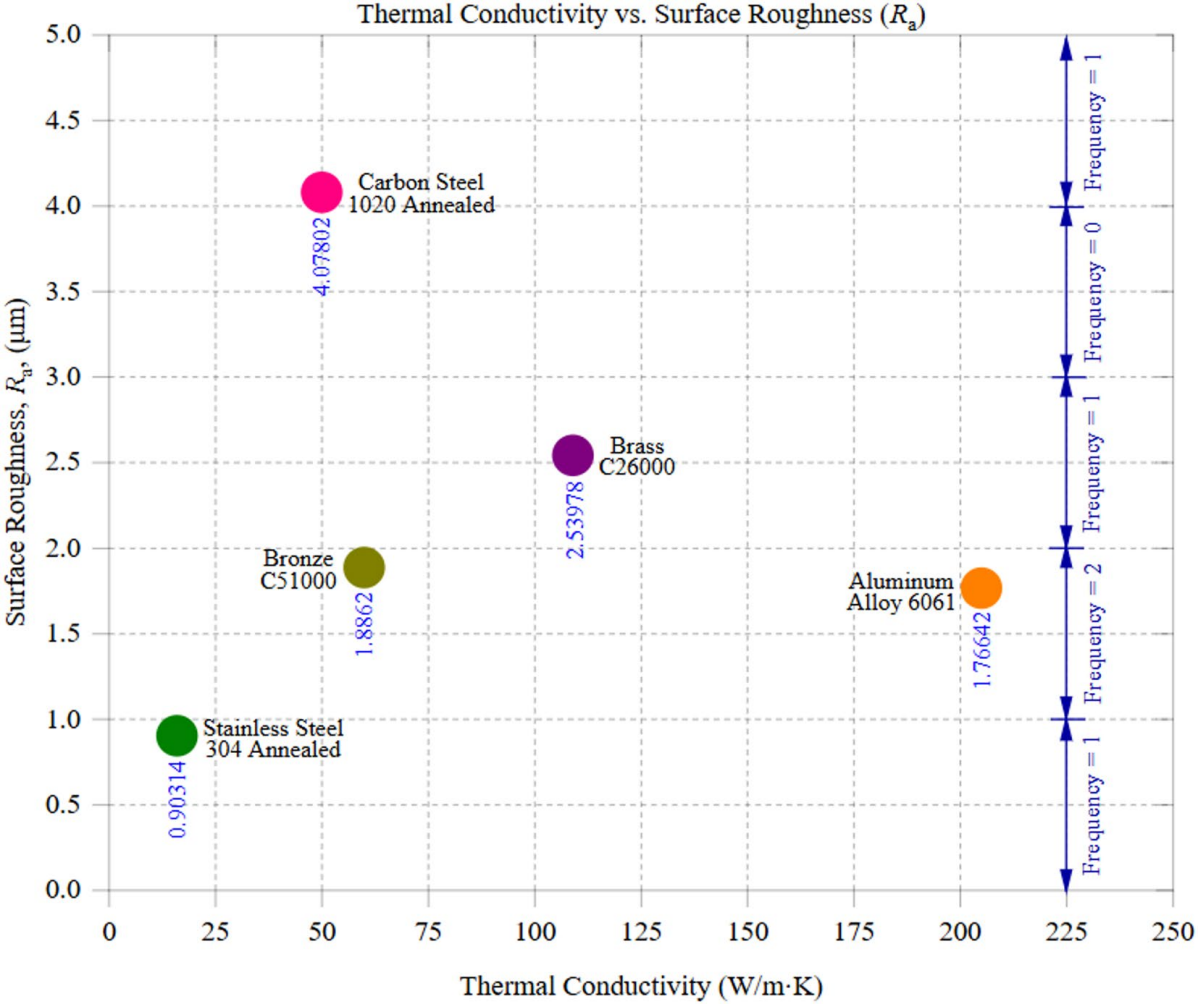
Thermal Conductivity vs. Surface Roughness (*R*_a_).

Figure 8 illustrates the relationship between thermal conductivity and the tested materials’ average surface roughness (*R*_a_), highlighting how a material’s ability to dissipate heat influences surface integrity. The trend suggests that materials with higher thermal conductivity generally yield smoother surfaces, as efficient heat dissipation minimizes localized thermal stresses, tool wear, and surface irregularities. Among the tested materials, aluminum alloy 6061, with the highest thermal conductivity of 205 W/m·K, exhibited an *R*_a_ of 1.77 μm with a standard deviation of 1.45 μm. Aluminum’s ability to dissipate heat efficiently ensures minimal temperature buildup at the cutting zone, reducing the likelihood of thermal expansion and tool wear. This property contributes to a relatively smooth surface, as rapid heat removal prevents the development of excessive residual stress. However, its surface roughness remains higher than expected for such a high-conductivity material, which may be attributed to built-up edge (BUE) formation. During machining, Aluminum’s ductility causes material adhesion to the tool, leading to irregular surface deposits and tearing effects, which can degrade the finish despite its thermal advantages. In contrast, carbon steel 1020, with a significantly lower thermal conductivity of 50 W/m·K, exhibited the highest surface roughness, with an *R*_a_ of approximately 4.08 μm (SD = 0.247 μm). This outcome aligns with the expectation that low thermal conductivity materials retain heat, leading to localized temperature spikes in the cutting zone. The inability to effectively dissipate heat accelerates tool wear, promotes oxidation effects, and increases plastic deformation, all of which contribute to a rougher machined surface. The observed roughness variability across different machining zones suggests that thermal softening and strain hardening effects occurred inconsistently across the workpiece. Intermediate materials, such as Brass C26000 (thermal conductivity = 109 W/m·K, *R*_a_ ≈ 2.54 μm, SD = 0.88 μm) and Bronze C51000 (thermal conductivity = 60 W/m·K, *R*_a_ ≈ 1.89 μm, SD = 1.441 μm), exhibited moderate surface roughness values. Brass, with its higher conductivity than carbon steel, dissipated heat more effectively, thereby reducing thermal stress buildup and enhancing the surface finish. However, Brass’s moderate hardness (~ 115 HBW) and tendency to exhibit microstructural anisotropy contributed to slight surface variations. Bronze, with a lower conductivity than Brass but a similar hardness (~ 112.5 HBW), produced a slightly smoother surface than Brass. This is likely due to Bronze’s improved chip-breaking characteristics, which lead to cleaner material removal and less burr formation during machining. An interesting deviation from the expected trend was observed in Stainless Steel 304, which has the lowest thermal conductivity of 16 W/m·K yet achieved an *R*_a_ of 0.90 μm (SD = 0.279 μm). A rougher surface would have been anticipated because poor thermal conductivity typically leads to higher residual stress accumulation, heat-affected zones, and increased cutting forces. However, Stainless Steel’s unique strain-hardening capability and the high cutting resistance of its austenitic microstructure contributed to a uniform chip formation process, likely resulting in a stable and consistent material removal mechanism. Stainless Steel’s work-hardening property also improves surface integrity, leading to a lower-than-expected *R*_a_ despite its thermal limitations. The results in Fig. 8 confirm that thermal conductivity plays a significant role in determining surface roughness during CNC machining, yet it does not act alone. While higher thermal conductivity materials, such as Aluminum, effectively dissipate heat and reduce tool wear, factors such as built-up edge formation, ductility, and adhesion effects can still influence the final surface finish. Lower thermal conductivity materials like carbon steel struggle with heat accumulation, leading to rougher finishes due to thermal expansion, work hardening, and accelerated tool degradation. The machining behavior of intermediate materials, such as Brass and Bronze, is dictated by a combination of thermal and mechanical properties, where chip formation stability and cutting resistance significantly impact surface quality.

The anomalous behavior of Stainless Steel 304, which shows the smoothest surface finish (*R*_a_ ≈ 0.9 μm, *W*_a_ ≈ 0.59 μm) despite having the lowest thermal conductivity (16 W/m·K), can be attributed to both metallurgical and tribological factors. Its strong strain-hardening ability enhances localized deformation resistance while cutting, leading to smoother shearing and less surface damage. The stable austenitic microstructure supports continuous chip formation with minimal vibration, reducing chatter and waviness. Furthermore, while stainless steel is thermally resistant, much of the cutting energy is dissipated via plastic deformation instead of being retained as residual heat. This energy redistribution lessens built-up edge (BUE) formation and stabilizes the interaction between the tool and workpiece. Together, these elements counterbalance the challenges posed by poor thermal conductivity, resulting in excellent surface integrity. This situation underscores the necessity of enhancing surface quality models to go beyond thermal conductivity and include microstructural effects and dynamic strain responses for more accurate predictions. Chip morphology observations further align with this trend: Carbon Steel 1020’s rougher surfaces were accompanied by unstable continuous chips with adhesion, while high-conductivity alloys like Brass produced smoother chips with fewer adhesion artifacts, reflecting the interplay of thermal dissipation and chip flow stability. It should be noted that no direct metallurgical analysis (such as grain size, micrographs, or phase characterization) was conducted in this study.

These findings emphasize the need for optimized cutting parameters tailored to each material’s thermal conductivity, hardness, and microstructural behavior. Controlling factors such as cutting speed, feed rate, and cooling techniques are crucial to mitigating heat-related surface deterioration and achieving high-quality finishes across different materials.

**Fig. 9:**
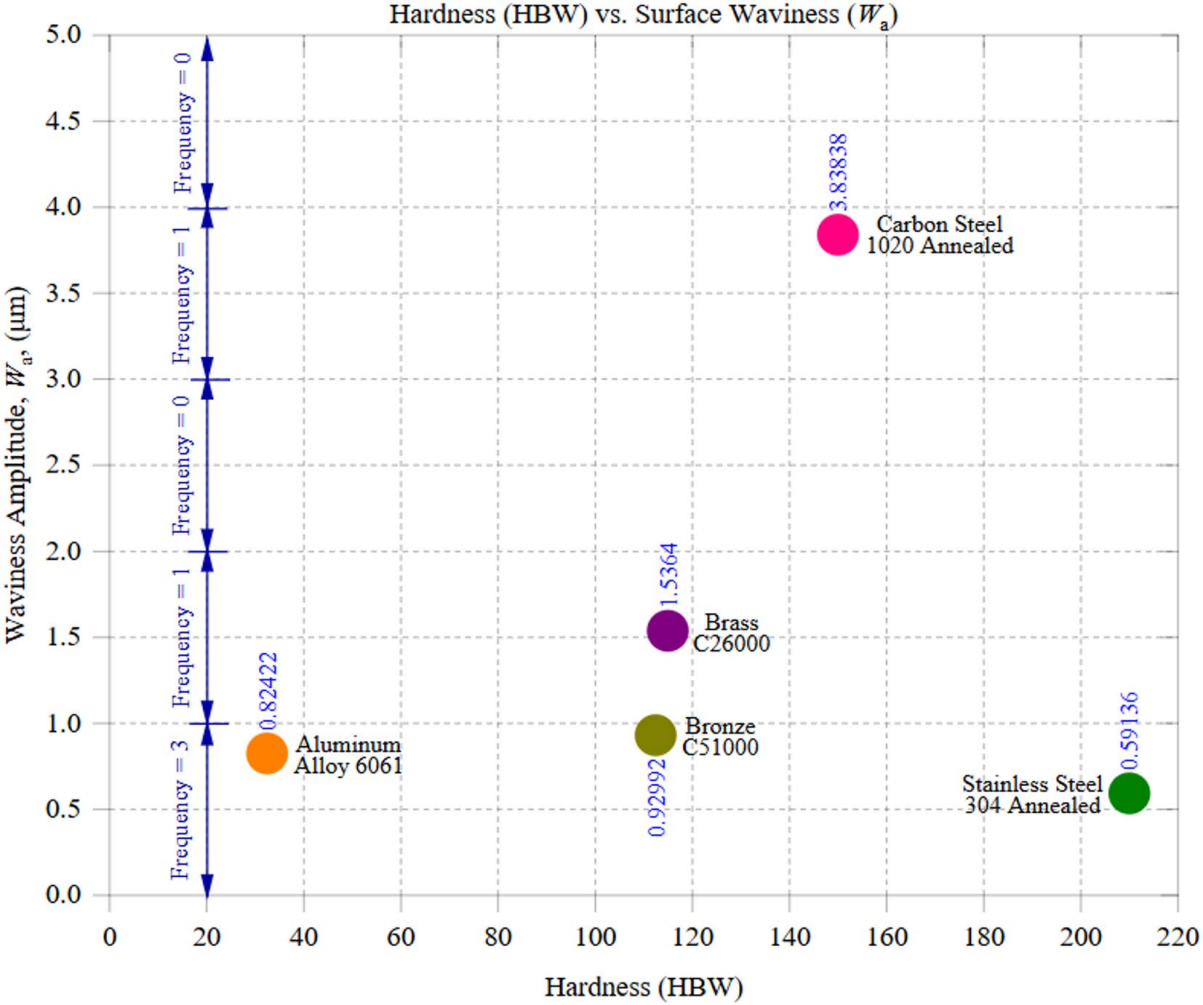
Hardness (HBW) vs. Waviness Amplitude (*W*_a_).

Figure 9 illustrates the relationship between material hardness (HBW) and surface waviness amplitude (*W*_a_), revealing how mechanical properties influence large-scale surface irregularities in CNC machining. The results indicate that harder materials do not necessarily exhibit higher waviness, as other factors, such as thermal conductivity, chip formation, and cutting resistance, play a significant role in surface generation. Among the tested materials, Stainless Steel 304, with the highest hardness of 210 HBW, exhibited the lowest *W*_a_ of 0.59 μm with a standard deviation of 0.18 μm. This result might initially seem counterintuitive, as harder materials typically generate greater cutting forces and surface deformations. However, Stainless Steel’s strain-hardening behavior and resistance to plastic deformation contribute to stable cutting conditions, minimizing waviness. Additionally, its low thermal conductivity (16 W/m·K) often leads to localized heating, which can cause thermal expansion and waviness in some cases. Still, in this instance, the controlled machining conditions likely mitigated these effects, resulting in a uniform surface. On the opposite end, Carbon Steel 1020, with a hardness of 150 HBW, exhibited the highest *W*_a_ of approximately 3.84 μm (SD = 0.64 μm). Unlike Stainless Steel, Carbon Steel’s ferritic-pearlitic microstructure and moderate thermal conductivity (50 W/m·K) likely contributed to more pronounced waviness. The reduced ability to dissipate heat efficiently leads to localized thermal softening, which can cause fluctuations in material removal. Additionally, increased tool wear and periodic chip formation inconsistencies contribute to amplified waviness. The higher standard deviation suggests greater variability in surface formation, reinforcing the influence of machining-induced thermal effects. Intermediate materials, such as Brass C26000 (115 HBW, *W*_a_ ≈ 1.54 μm, SD = 0.53 μm) and Bronze C51000 (112.5 HBW, *W*_a_ ≈ 0.93 μm, SD = 0.78 μm), exhibited moderate surface waviness values. Brass, with its relatively high thermal conductivity (109 W/m·K), dissipates heat more effectively than carbon steel, reducing localized expansion and contraction effects that contribute to waviness. However, its ductility and tendency to exhibit strain localization during machining can introduce surface undulations, leading to slightly higher waviness than Bronze. Bronze, with a lower conductivity (60 W/m·K) but similar hardness, demonstrated improved waviness control, likely due to better chip fragmentation and stable cutting behavior. The moderate hardness of both materials contributes to a balanced cutting response, reducing excessive tool deflection and chip adhesion. Aluminum alloy 6061 has an intermediate *W*_a_ of 0.82 μm (SD = 0.79 μm), even though it has the lowest hardness of 95 HBW. Its high thermal conductivity (205 W/m·K) effectively dissipates heat, preventing excessive thermal stresses that could increase waviness. However, Aluminum’s soft nature makes it susceptible to elastic deformation, particularly at higher feed rates, which may contribute to localized waviness inconsistencies. Additionally, built-up edge (BUE) formation and material adhesion to the tool can introduce microscopic irregularities, which result in non-uniform waviness patterns when accumulated across larger zones.

The observed trends reinforce that hardness alone does not dictate surface waviness. While harder materials, such as stainless steel, benefit from stable chip formation and controlled plastic deformation, reducing waviness, others, such as carbon steel, experience amplified waviness due to increased tool wear and localized heat effects. Similarly, softer materials like aluminum exhibit moderate waviness due to their susceptibility to adhesion effects and elastic recovery. This observation is also supported by the standard deviation values, which show that materials with higher thermal conductivity (like Aluminum and Brass) tend to have less variation in *W*_a_, and materials with lower thermal conductivity (like carbon steel) have more variation.

These findings emphasize that hardness and thermal conductivity influence surface waviness, but machining parameters and tool interactions are crucial in controlling deviations. Optimizing cutting speeds, feed rates, and tool geometries is essential for minimizing waviness and achieving superior surface quality across different materials.

**Fig. 10:**
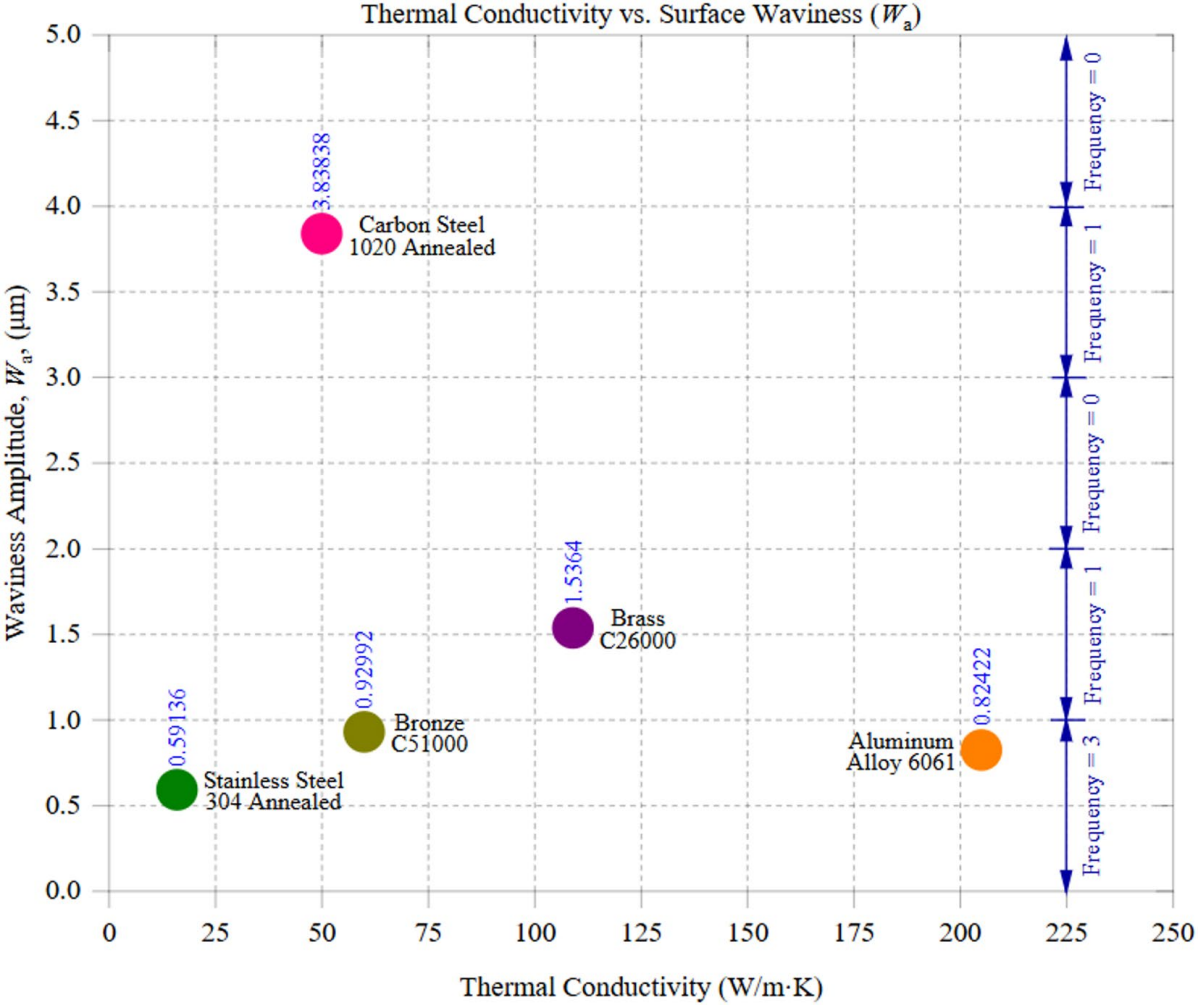
Thermal Conductivity vs. Waviness Amplitude (*W*_a_).

Figure 10 illustrates the relationship between thermal conductivity and surface waviness amplitude (*W*_a_), demonstrating how a material’s heat dissipation capability influences large-scale surface irregularities. The data reveal a consistent trend: materials with higher thermal conductivity exhibit lower surface waviness, while those with poor heat dissipation tend to develop more pronounced undulations. This correlation underscores the role of thermal management in machining stability and surface quality. Among the tested materials, Aluminum alloy 6061, with the highest thermal conductivity of 205 W/m·K, exhibited the lowest *W*_a_ of 0.82 μm, with a standard deviation of 0.79 μm. Aluminum’s exceptional heat dissipation prevents excessive temperature buildup at the cutting interface, ensuring stable tool engagement and uniform material removal. This effective heat management reduces the risk of thermal deformation, tool wear, and excessive cutting forces, which collectively contribute to a smoother waviness profile. However, Aluminum’s high ductility and adhesion tendency may lead to minor inconsistencies in waviness, especially at higher feed rates, where built-up edge (BUE) formation affects chip detachment. On the opposite end, Carbon Steel 1020, with a low thermal conductivity of 50 W/m·K, exhibited the highest *W*_a_ of 3.84 μm (SD = 0.58 μm). The inability to dissipate heat efficiently results in localized temperature buildup, which, in turn, softens the material in certain regions while hardening others through strain-hardening effects. This thermal imbalance contributes to cutting force variations, inconsistent chip formation, and tool wear acceleration, leading to severe surface undulations. The higher standard deviation in *W*_a_ suggests that carbon steel’s surface irregularities fluctuate significantly across different machining zones, further emphasizing the impact of thermal constraints on machining stability. Intermediate materials, such as Brass C26000 (thermal conductivity = 109 W/m·K, *W*_a_ ≈ 1.54 μm, SD = 0.53 μm) and Bronze C51000 (thermal conductivity = 60 W/m·K, *W*_a_ ≈ 0.93 μm, SD = 0.79 μm), exhibited moderate surface waviness. Brass, with its superior heat conduction compared to carbon steel, dissipates heat more effectively, thereby reducing thermal stress accumulation. However, its moderate hardness and microstructural composition contribute to localized waviness variations, particularly in inconsistent chip formation zones. Bronze, with lower conductivity than Brass but slightly higher hardness, demonstrated a relatively smoother waviness profile, likely due to its improved chip-breaking characteristics, which minimize excessive tool-material interaction forces. An unexpected deviation from the expected trend was observed in Stainless Steel 304, which, despite having the lowest thermal conductivity of 16 W/m·K, exhibited a *W*_a_ of 0.59 μm (SD = 0.18 μm). A rougher waviness profile would have been anticipated because poor thermal conductivity generally leads to higher residual stress and thermal deformation. However, Stainless Steel’s strain-hardening ability and work-hardening behavior contribute to a consistent cutting response, reducing surface irregularities. Its high cutting resistance stabilizes chip formation, preventing erratic tool engagement and ensuring a uniform waviness profile. The lower standard deviation further indicates greater consistency in its surface formation compared to other materials.

These findings confirm that thermal conductivity is a key determinant of surface waviness in CNC machining. High thermal conductivity materials like Aluminum effectively dissipate heat, minimizing residual stress accumulation and maintaining a smooth waviness profile. Conversely, low thermal conductivity materials like Carbon Steel experience heat buildup, which promotes surface undulations due to uneven material softening and increased tool wear. The standard deviation values further emphasize the influence of thermal behavior on machining consistency, where materials with better heat dissipation (Aluminum and Brass) show lower variability in *W*_a_. In contrast, materials with poor heat conduction (Carbon Steel) exhibit higher fluctuations. This reinforces the importance of optimizing cutting speeds, cooling strategies, and tool geometries to mitigate thermal effects and achieve superior surface consistency across different materials.

Table 4 presents a sensitivity analysis of surface roughness (*R*_a_) in relation to ± 10% changes in thermal conductivity for five different engineering materials. It should be noted that sensitivity analysis was confined to *R*_a_. Waviness (*W*_a_) was summarized descriptively (mean/SD/CV) because its long-wavelength features are dominated by system-level dynamics (machine vibrations, fixturing, tool-holder compliance) and are highly sensitive to ISO filtering cut-offs. Under the present unified conditions, *W*_a_ shows limited responsiveness to small material-property perturbations (± 10%), so elasticity estimates would risk being confounded and non-informative. The findings reveal a consistent and symmetric pattern: enhancing thermal conductivity by 10% decreased *R*_a_ by 5.8% to 6.3%, while a 10% reduction in conductivity produced a comparable increase. This range distinctly illustrates a clear, proportional, and inverse relationship between thermal conductivity and surface roughness, all while maintaining constant machining conditions.

The material’s heat dissipation during cutting dictates this response. High-conductivity materials such as Aluminum 6061 (205 W/m·K) and Brass C26000 (109 W/m·K) effectively transfer heat away from the tool-workpiece interface. This action reduces thermal softening, limits built-up edge (BUE) development, and aids in stable chip separation, ultimately improving surface quality. Conversely, low-conductivity materials like Stainless Steel 304 (16 W/m·K) and Carbon Steel 1020 (50 W/m·K) retain more heat in the cutting zone, which leads to faster tool wear and increased surface roughness.

While absolute *R*_a_ values vary because of factors like hardness, chip morphology, and microstructural influences, the relative sensitivity to thermal conductivity is consistent among all materials. This highlights the importance of thermal transport properties as a crucial factor in surface integrity during CNC turning. These insights serve as a valuable guide for predicting surface roughness changes caused by variations in thermal properties stemming from differences in alloy composition or material variability between batches.

**Table 4:** Sensitivity of surface roughness (*R*_a_) to ± 10% variation in thermal conductivity for five engineering materials under identical machining conditions.

Material	Thermal Conductivity (W/m·K)	*R*_a_ Baseline (µm)	*R*_a_ at + 10% Conductivity (µm)	*R*_a_ at −10% Conductivity (µm)
Aluminum Alloy 6061	205	1.77	1.6106	1.9692
Brass C26000	109	2.54	2.3094	2.8216
Bronze C51000	60	1.89	1.7173	2.1000
Stainless Steel 304 Annealed	16	0.90	0.8182	1.0000
Carbon Steel 1020 Annealed	50	4.08	3.7091	4.5333

The sensitivity results presented in Table 4 indicate a consistent linear and proportional trend: a ± 10% change in thermal conductivity leads to a variation in *R*_a_ of roughly 5.8–6.3%, irrespective of the material type. This consistent behavior underscores a predictive rule that is applicable across various alloys with different machinability characteristics. Instead of reiterating established thermal effects, this analysis facilitates the prediction of surface roughness changes that emerge from variations in thermal properties, including batch differences or compositional alterations. By analyzing these relationships under the same conditions, the model serves as a valuable forecasting tool for surface preparation and material replacement in precision machining.

### Profile uniformity analysis (*R*_q_/*R*_a_, *W*_q_/*W*_a_, *R*_t_/*R*_z_, *W*_t_/*W*_z_)

**Fig. 11:**
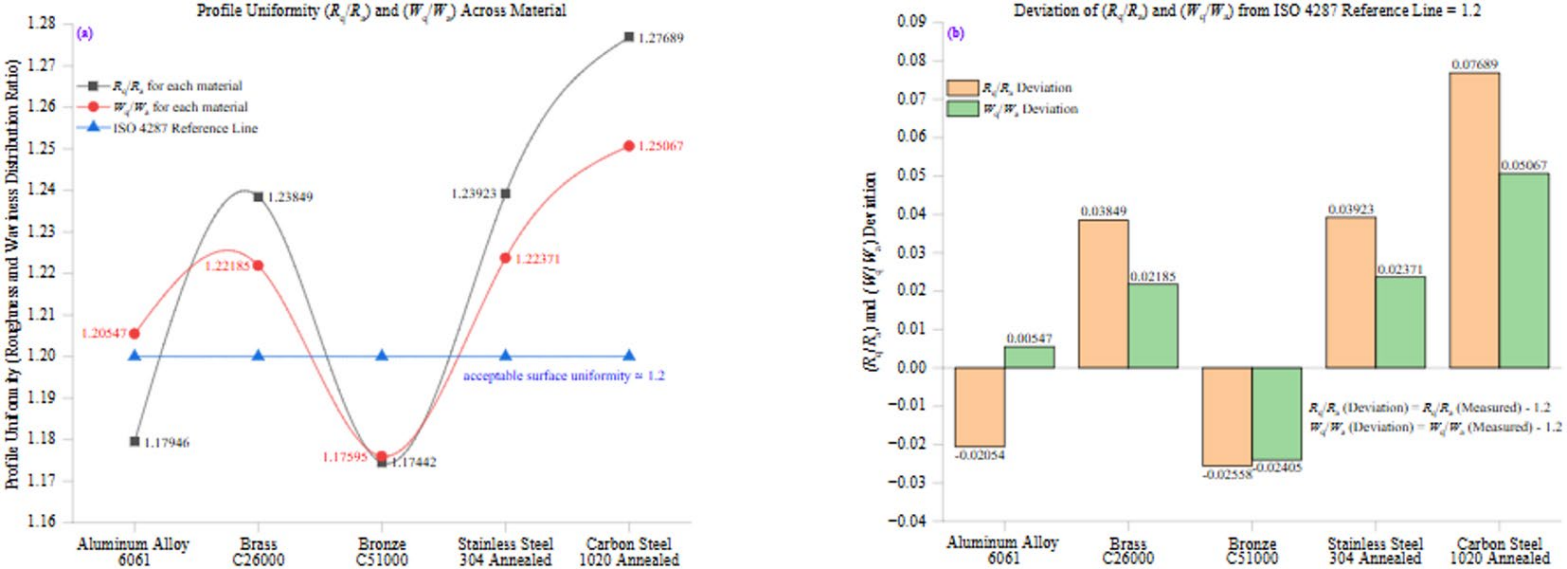
**(a)** Profile Uniformity (*R*_q_/*R*_a_) and (*W*_q_/*W*_a_) Across Material **(b)** Deviation of (*R*_q_/*R*_a_) and (*W*_q_/*W*_a_) From ISO 4287 Reference Line = 1.2.

Figure 11 presents a two-part analysis of profile uniformity across all tested materials, focusing on the ratios of surface roughness (*R*_q_/*R*_a_) and waviness (*W*_q_/*W*_a_). Figure 11(a) illustrates the variation of these ratios among different materials. In contrast, Fig. 11(b) highlights their deviation from the ISO 4287 reference value of 1.2, representing the expected theoretical uniformity in well-machined surfaces.

In Fig. 11(a), materials with higher thermal conductivity and lower hardness tend to exhibit lower *R*_q_/*R*_a_ and *W*_q_/*W*_a_ ratios, indicating a more uniform surface texture and improved machining consistency. Aluminum alloy 6061, which has the highest thermal conductivity (205 W/m·K) and lowest hardness (95 HBW), exhibited the lowest ratios, with *R*_q_/*R*_a_ at 1.18 and *W*_q_/*W*_a_ at 1.21. This suggests that Aluminum’s high heat dissipation capacity minimizes localized temperature fluctuations at the cutting interface, preventing excessive tool wear and ensuring a stable cutting process with minimal surface roughness and waviness deviations. In contrast, Stainless Steel 304, which has the lowest thermal conductivity (16 W/m·K) and the highest hardness (210 HBW), exhibited the highest profile uniformity ratios, with *R*_q_/*R*_a_ at 1.24 and *W*_q_/*W*_a_ at 1.22. The poor heat dissipation characteristics of stainless steel result in localized thermal expansion and contraction, increasing the risk of cutting tool deflection and surface irregularities. The increased cutting resistance leads to greater tool wear and variability in material removal, resulting in a less uniform machined surface. Intermediate materials, such as Brass C26000 (109 W/m·K) and Bronze C51000 (60 W/m·K), exhibited moderate profile uniformity ratios. Brass recorded an *R*_q_/*R*_a_ of 1.24 and *W*_q_/*W*_a_ of 1.22, while Bronze exhibited slightly lower values of *R*_q_/*R*_a_ at 1.17 and *W*_q_/*W*_a_ at 1.18. The differences between these materials can be attributed to their distinct hardness values (Brass: 115 HBW, Bronze: 112.5 HBW) and microstructural differences. Brass’s higher thermal conductivity allows it to dissipate heat more efficiently, which reduces thermal stress and surface variability. However, its ductility and tendency for localized plastic deformation may contribute to minor profile variations. Despite its lower thermal conductivity, Bronze exhibits superior cutting stability due to its high wear resistance, which contributes to slightly lower roughness and waviness ratios. Carbon steel 1020, with a thermal conductivity of 50 W/m·K and hardness of 150 HBW, exhibited ratios slightly higher than Aluminum but lower than stainless steel, with *R*_q_/*R*_a_ at 1.28 and *W*_q_/*W*_a_ at 1.25. The moderate values reflect carbon steel’s balanced machining characteristics, where its combination of thermal and mechanical properties results in stable but slightly variable surface finishes. The higher-than-expected profile uniformity ratios indicate that heat buildup at the cutting zone may have led to increased tool wear, contributing to minor inconsistencies in surface texture formation.

Figure 11(b) highlights the deviation of these ratios from the ISO 4287 reference line of 1.2, representing the ideal uniformity value for well-machined surfaces. The values of materials like Aluminum and Bronze are closer to the reference, which means their machining processes produce smoother, uniform surfaces with minimal deviation. Conversely, Stainless Steel and Carbon Steel deviate significantly from the reference line, indicating greater surface profile variability. The elevated ratios for stainless steel confirm the challenges associated with machining low thermal conductivity materials, where localized heat accumulation leads to increased roughness and waviness inconsistencies. These findings underscore the importance of material selection and machining parameter optimization in controlling surface uniformity. Materials with superior thermal conductivity and moderate hardness, such as Aluminum and Bronze, consistently achieve better surface uniformity. In contrast, harder and less thermally conductive materials require stricter process control to minimize variability. The deviation from the ISO 4287 reference line further reinforces the need for fine-tuned cutting strategies, such as adaptive feed rates, optimized cooling methods, and appropriate tool coatings, to enhance machining consistency.

**Fig. 12:**
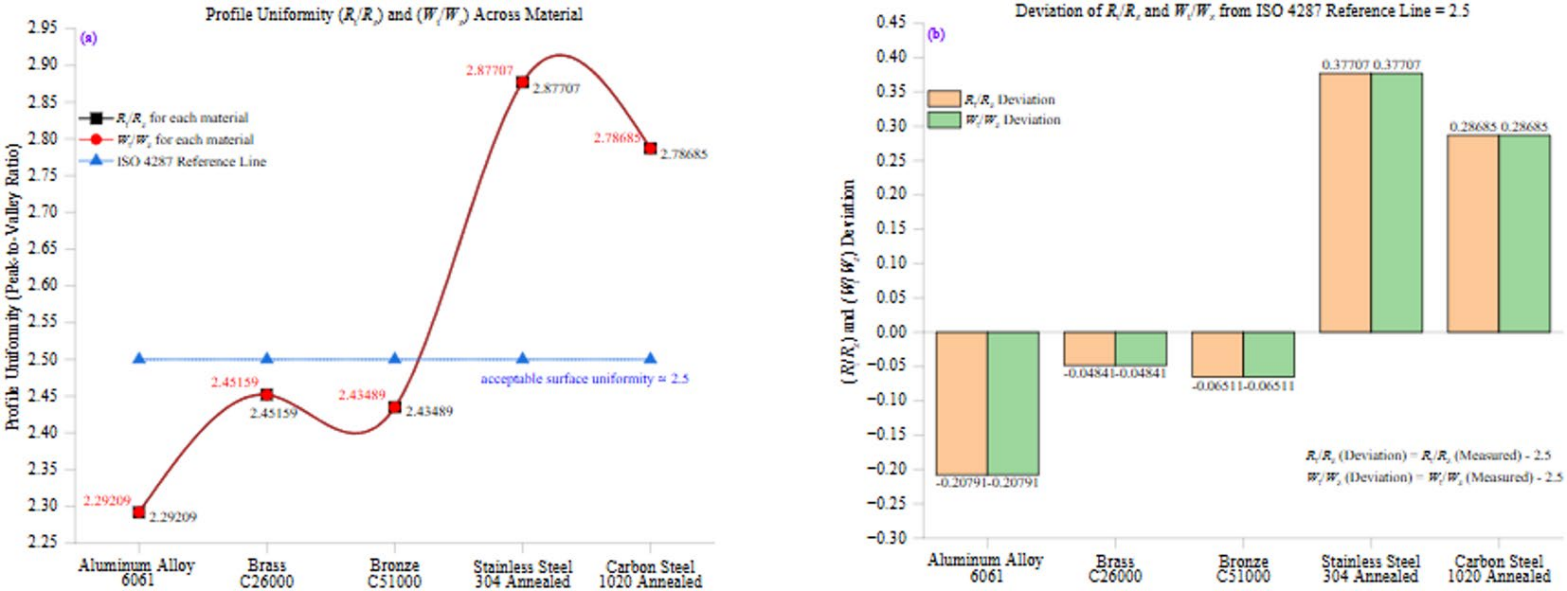
**(a)** Profile Uniformity (*R*_t_/*R*_z_) and (*W*_t_/*W*_z_) Across Material **(b)** Deviation of (*R*_t_/*R*_z_) and (*W*_t_/*W*_z_) From ISO 4287 Reference Line = 2.5.

Figure 12 presents a comparative analysis of profile uniformity across different materials, focusing on the ratios of total surface roughness (*R*_t_/*R*_z_) and total surface waviness (*W*_t_/*W*_z_). Figure 12(a) illustrates the variation of these ratios among tested materials. In contrast, Fig. 12(b) highlights their deviation from the ISO 4287 reference value of 2.5, which serves as the theoretical benchmark for well-machined surfaces.

In Fig. 12(a), materials with higher thermal conductivity and lower hardness tend to exhibit lower *R*_t_/*R*_z_ and *W*_t_/*W*_z_ ratios, indicating a more uniform peak-to-valley distribution and consistent material removal. Aluminum alloy 6061, with its high thermal conductivity (205 W/m·K) and low hardness (95 HBW), recorded the lowest *R*_t_/*R*_z_ (2.29) and *W*_t_/*W*_z_ (2.29). This suggests that Aluminum’s excellent heat dissipation ability minimizes temperature-induced stress variations, thereby maintaining cutting stability and preventing excessive peak formation on the surface and waviness profile. The relatively low deviation in peak-to-valley characteristics confirms its suitability for high-precision machining applications. Conversely, Stainless Steel 304, characterized by poor thermal conductivity (16 W/m·K) and high hardness (210 HBW), exhibited the highest ratios, with *R*_t_/*R*_z_ at 2.88 and *W*_t_/*W*_z_ at 2.87. The material’s low thermal dissipation capability results in localized heat accumulation, which enhances cutting tool deflection and promotes irregular material removal. The high cutting resistance also contributes to significant fluctuations in peak height distribution, leading to an increased *R*_t_/*R*_z_ ratio. The higher-than-expected *W*_t_/*W*_z_ ratio suggests that waviness peaks and valleys are significantly impacted by inconsistent thermal expansion and contraction during machining. Intermediate materials, such as Brass C26000 (109 W/m·K) and Bronze C51000 (60 W/m·K), exhibit moderate peak-to-valley uniformity ratios. Brass recorded an *R*_t_/*R*_z_ of 2.45 and *W*_t_/*W*_z_ of 2.45, while Bronze displayed slightly lower values, with *R*_t_/*R*_z_ at 2.43 and *W*_t_/*W*_z_ at 2.43. The differences between these materials follow the trend of their distinct hardness levels (Brass: 115 HBW, Bronze: 112.5 HBW) and machinability properties. Due to its higher thermal conductivity, Brass exhibits superior heat dissipation, reducing fluctuations in peak formation. However, its ductility and tendency for minor plastic deformation during machining contribute to slight deviations in total surface roughness and waviness ratios. Conversely, Bronze exhibits superior cutting stability, leading to a more consistent peak structure in roughness and waviness profiles. Carbon steel 1020, with moderate thermal conductivity (50 W/m·K) and hardness (150 HBW), exhibited slightly elevated ratios of *R*_t_/*R*_z_ (2.79) and *W*_t_/*W*_z_ (2.78). This is due to its moderate cutting resistance, which leads to uneven tool wear, resulting in non-uniform peak distributions. Although its heat dissipation is superior to that of Stainless Steel, its surface profile variability remains higher than that of Brass and Aluminum.

Figure 12(b) highlights the deviation of these ratios from the ISO 4287 reference value of 2.5, representing an ideal uniformity benchmark for peak-to-valley distribution. Materials such as Aluminum and Bronze, which exhibit values close to the reference line, confirm their machining stability, resulting in highly controlled peak-to-valley consistency. In contrast, stainless steel and carbon steel display significant deviations from the reference, confirming greater variability in their peak formations. The increased *R*_t_/*R*_z_ and *W*_t_/*W*_z_ ratios for these materials indicate machining-induced irregularities caused by inadequate heat dissipation, material-specific work hardening, and tool wear effects. These findings reinforce the importance of material selection and machining parameter control in managing peak-to-valley uniformity. Materials with higher thermal conductivity and moderate hardness, such as Aluminum and Bronze, consistently demonstrate smoother peak structures with minimal fluctuations, whereas harder and thermally resistant materials require enhanced cooling strategies and adaptive feed control to mitigate profile inconsistencies. The observed deviations from the ISO 4287 reference standard further highlight the necessity of optimizing machining conditions, including cutting speeds, feed rates, and tool coatings, to achieve superior surface integrity and minimize variability in peak formations.

### Surface and Waviness Skewness/Kurtosis (*R*_sk_, *R*_ku_, *W*_sk_, *W*_ku_)

**Fig. 13:**
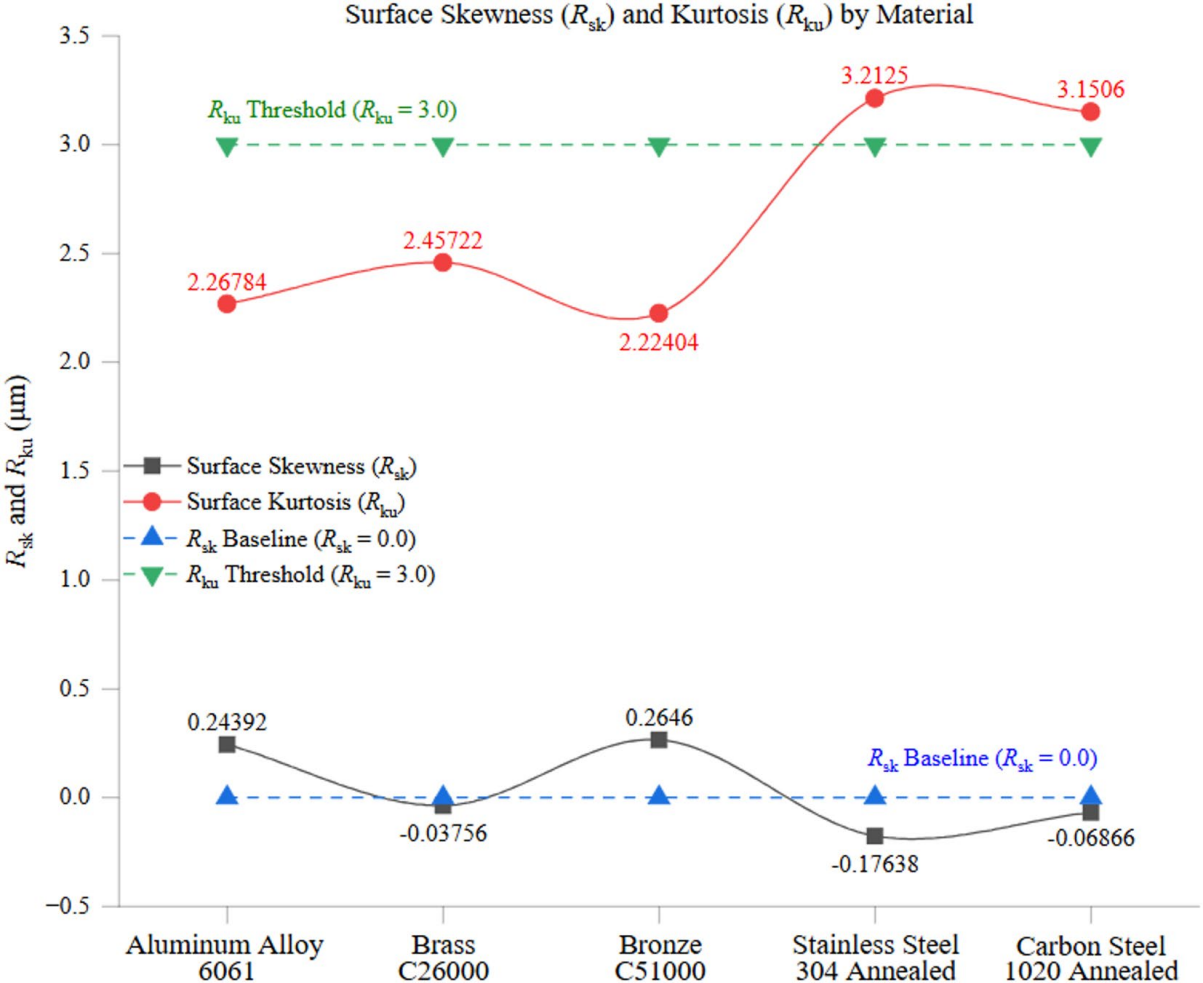
Surface Skewness (*R*_sk_) and Kurtosis (*R*_ku_) by Material.

Figure 13 presents the surface skewness (*R*_sk_) and kurtosis (*R*_ku_) values for the machined materials, offering critical insights into the statistical distribution of peak and valley structures. These parameters characterize the fine-scale texture of a surface, where skewness (*R*_sk_) indicates the asymmetry of height distributions, and kurtosis (*R*_ku_) describes the sharpness or flatness of surface peaks. Among the tested materials, Brass C26000 exhibited the most symmetrical surface profile, with an *R*_sk_ value of approximately − 0.04 and an *R*_ku_ value of 2.46. The near-zero skewness suggests a balanced distribution of peaks and valleys. In contrast, the kurtosis value of Brass C26000 (*R*_ku_ = 3.0) is slightly below the reference Gaussian distribution, which indicates a relatively broad but evenly distributed surface texture. These results align well with Brass’s high thermal conductivity (109 W/m·K) and moderate hardness (115 HBW), which contribute to stable heat dissipation and consistent material removal, reducing the likelihood of extreme peaks or valleys. Bronze C51000 exhibited a slightly positive *R*_sk_ value of 0.26, indicating a tendency for sharper peaks rather than deep valleys. Its *R*_ku_ value of 2.22 suggests a broader, less-peaked surface profile, which reflects its lower thermal conductivity (60 W/m·K) and higher hardness (112.5 HBW). The positive skewness may be attributed to localized material deformation and adhesion effects during machining, while the reduced kurtosis value suggests a relatively uniform surface without dominant asperities. Stainless Steel 304, with an *R*_sk_ value of −0.18 and an *R*_ku_ value of 3.21, exhibited a profile dominated by shallow valleys with occasional sharp peaks. The negative skewness indicates a more significant presence of depressions rather than protrusions, which is typical of materials with high hardness (210 HBW) and poor thermal conductivity (16 W/m·K). These properties contribute to increased tool wear, localized thermal stresses, and material hardening, leading to variations in surface morphology. The slightly elevated kurtosis suggests that sharp, isolated peaks exist on the surface, likely due to inconsistencies in chip formation or microscopic tool deflections during machining.

Carbon Steel 1020 recorded an *R*_sk_ value of −0.07, closely resembling a symmetric surface profile, with an *R*_ku_ value of 3.15. The moderately negative skewness and kurtosis slightly exceeding 3.0 suggest a surface with a balanced peak-valley distribution but a tendency toward sharper localized asperities. Its intermediate thermal conductivity (50 W/m·K) and hardness (150 HBW) contribute to this behavior, placing its surface characteristics between softer materials (such as Aluminum and Brass) and harder materials (such as Stainless Steel). These results indicate that Carbon Steel maintains a relatively uniform texture but is prone to forming slightly more distinct peaks due to its thermal expansion behavior under machining conditions. Aluminum alloy 6061 exhibited a slightly positive *R*_sk_ value of 0.24 and an *R*_ku_ value of 2.27, indicating a surface profile with sharper peaks and a broader distribution of asperities. Aluminum’s high thermal conductivity (205 W/m·K) and low hardness (95 HBW) typically promote smooth surface finishes; however, the presence of positive skewness suggests a tendency for minor material buildup at the surface, potentially due to adhesion effects or built-up edge (BUE) formation during machining. Furthermore, the relatively low kurtosis confirms a broad, stable distribution of surface features rather than extreme peaks or valleys.

These findings emphasize the profound impact of material properties on surface feature distribution. Softer materials with higher thermal conductivity, such as Aluminum and Brass, generally yield smoother, more uniform textures. In contrast, harder and thermally resistant materials, like Stainless Steel and Carbon Steel, tend to exhibit greater variability in their peak-valley structures. The negative skewness and higher kurtosis observed in harder materials suggest a tendency for deep valleys and localized asperities, which can negatively influence functional performance in applications requiring high precision and wear resistance. These results reinforce the necessity of tailoring machining parameters to specific material properties to minimize surface irregularities and achieve optimal functional performance.

Beyond conventional regression and coefficient of variation analysis, this study incorporates higher-order statistical moments, skewness (*R*_sk_, *W*_sk_) and kurtosis (*R*_ku_, *W*_ku_), to assess asymmetry and sharpness in both surface and waviness distributions. These shape descriptors provide insights into surface uniformity and defect-prone tendencies, with positive skewness indicating peak-dominated profiles and higher kurtosis signaling concentrated asperity zones. The inclusion of interquartile range (IQR) further strengthens the statistical depth by quantifying variability around the median, which is less sensitive to outliers than standard deviation. Additionally, the sensitivity analysis presented in Table 4 can be interpreted as a bounded one-factor-at-a-time (OFAT) study, enabling the predictive estimation of surface roughness shifts resulting from changes in thermal conductivity. Collectively, these methods support a more robust interpretation of the data beyond classical mean–variance models.

**Fig. 14:**
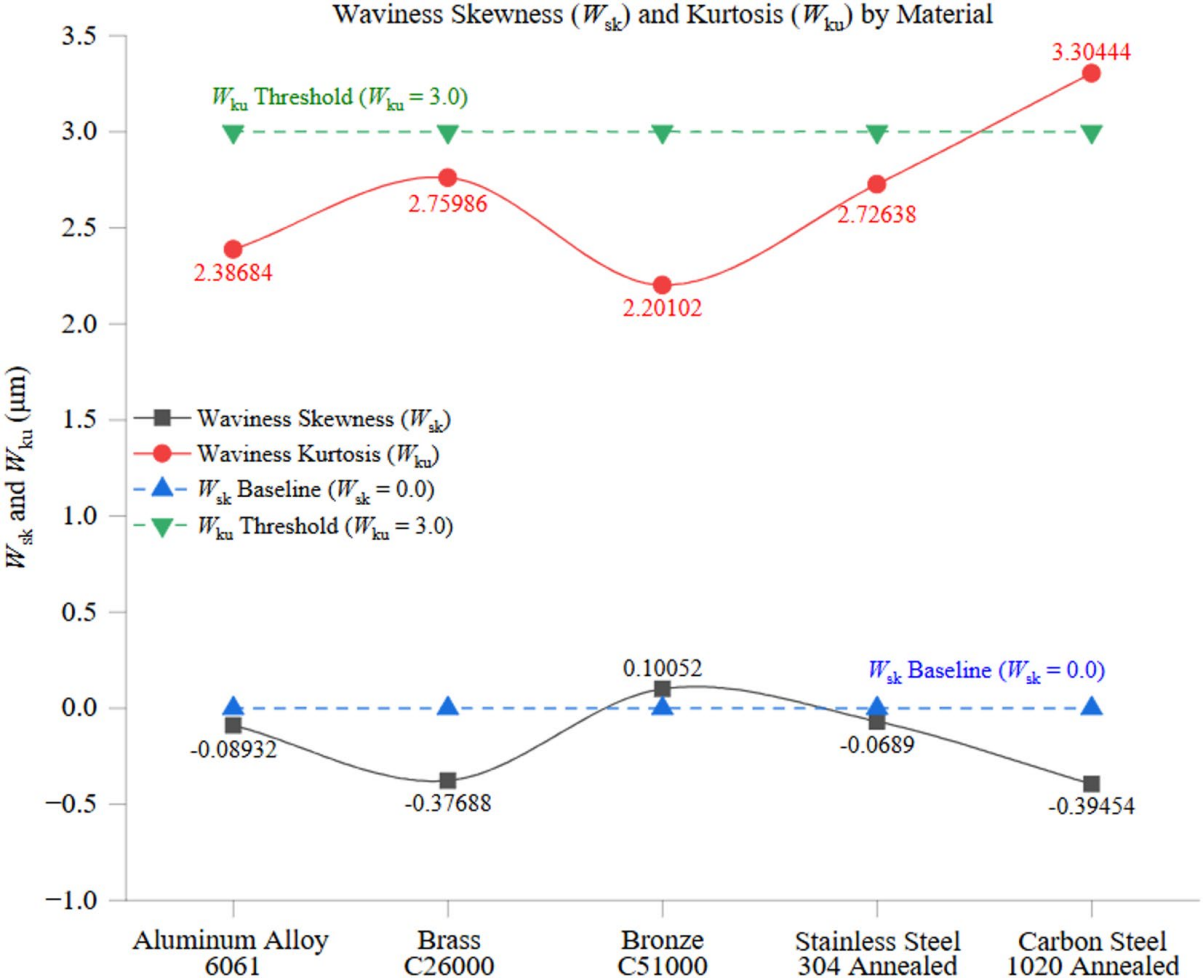
Waviness Skewness (*W*_sk_) and Kurtosis (*W*_ku_) by Material.

Figure 14 presents the waviness skewness (*W*_sk_) and kurtosis (*W*_ku_) values for the machined materials, providing insights into the statistical distribution of large-scale surface features. These parameters are critical for understanding the formation of macro-scale surface undulations and their impact on functional performance. Skewness (*W*_sk_) describes the asymmetry of the waviness distribution, whereas kurtosis (*W*_ku_) indicates the sharpness or flatness of the waviness peaks and valleys. Among the tested materials, bronze C51000 exhibited a slightly positive *W*_sk_ value (0.10), indicating a surface profile where peaks are slightly more pronounced than valleys. Its *W*_ku_ value of 2.20, which is slightly below the Gaussian reference of 3.0, suggests a broad waviness distribution with relatively mild surface undulations. These characteristics are influenced by Bronze’s intermediate thermal conductivity (60 W/m·K) and hardness (112.5 HBW), which facilitate stable machining but still allow for moderate surface irregularities due to variations in cutting resistance. Stainless Steel 304 exhibited a slightly negative *W*_sk_ value (−0.07) and a *W*_ku_ value of 2.73, slightly below 3.0. These results indicate a waviness profile dominated by shallow valleys rather than sharp peaks, with a relatively balanced distribution of undulations. Given stainless steel’s low thermal conductivity (16 W/m·K) and high hardness (210 HBW), these findings suggest that localized thermal stress and high cutting resistance introduce minor waviness irregularities but do not create extreme surface distortions. The results also imply that while stainless steel’s machining characteristics contribute to some minor surface deformation, the overall waviness remains controlled under the given cutting conditions. Aluminum alloy 6061 displayed a near-symmetrical waviness profile, with a negative *W*_sk_ value of −0.09 and a *W*_ku_ value of 2.39. These results indicate a balanced surface texture with minimal sharp peaks or valleys, contributing to a relatively uniform waviness distribution. The high thermal conductivity (205 W/m·K) and low hardness (95 HBW) of Aluminum allow for efficient heat dissipation and stable cutting conditions, minimizing fluctuations in surface waviness. The absence of extreme peaks or valleys suggests that Aluminum’s favorable machinability contributes to maintaining a uniform surface over a larger scale. Brass C26000 recorded a more pronounced negative *W*_sk_ value (−0.38) and a *W*_ku_ value of 2.76, suggesting a waviness profile with deeper valleys and sharper macro-scale undulations than Aluminum or Stainless Steel. These characteristics can be linked to Brass’s relatively high thermal conductivity (109 W/m·K) and moderate hardness (115 HBW), facilitating efficient heat dissipation but allowing for some tool-material interactions contributing to localized waviness formation. The presence of deeper valleys may indicate intermittent material adhesion effects or periodic tool deflections that introduce minor macro-scale inconsistencies in the surface profile. Carbon Steel 1020 exhibited the most pronounced deviations, with a *W*_sk_ value of −0.39 and the highest *W*_ku_ value of 3.30, indicating a surface profile dominated by deeper valleys and a highly peaked waviness distribution. This behavior reflects carbon steel’s lower thermal conductivity (50 W/m·K) and higher hardness (150 HBW), which increase cutting resistance and induce greater thermal stress. As a result, higher tool wear and unstable cutting conditions contribute to significant waviness irregularities, with localized depressions and sharper macro-scale peaks. The high kurtosis value suggests that waviness peaks are more concentrated, leading to rougher large-scale surface morphology.

These findings emphasize the substantial influence of material properties on waviness distributions. Materials with higher thermal conductivity and lower hardness, such as Aluminum alloy 6061, tend to produce more stable, symmetric waviness profiles. In contrast, harder, less conductive materials like carbon steel 1020 generate more pronounced surface undulations due to increased cutting forces and thermal stresses. These results highlight the importance of optimizing machining parameters to control waviness and improve surface consistency, particularly for materials prone to thermal distortions and cutting-induced irregularities.

**Fig. 15:**
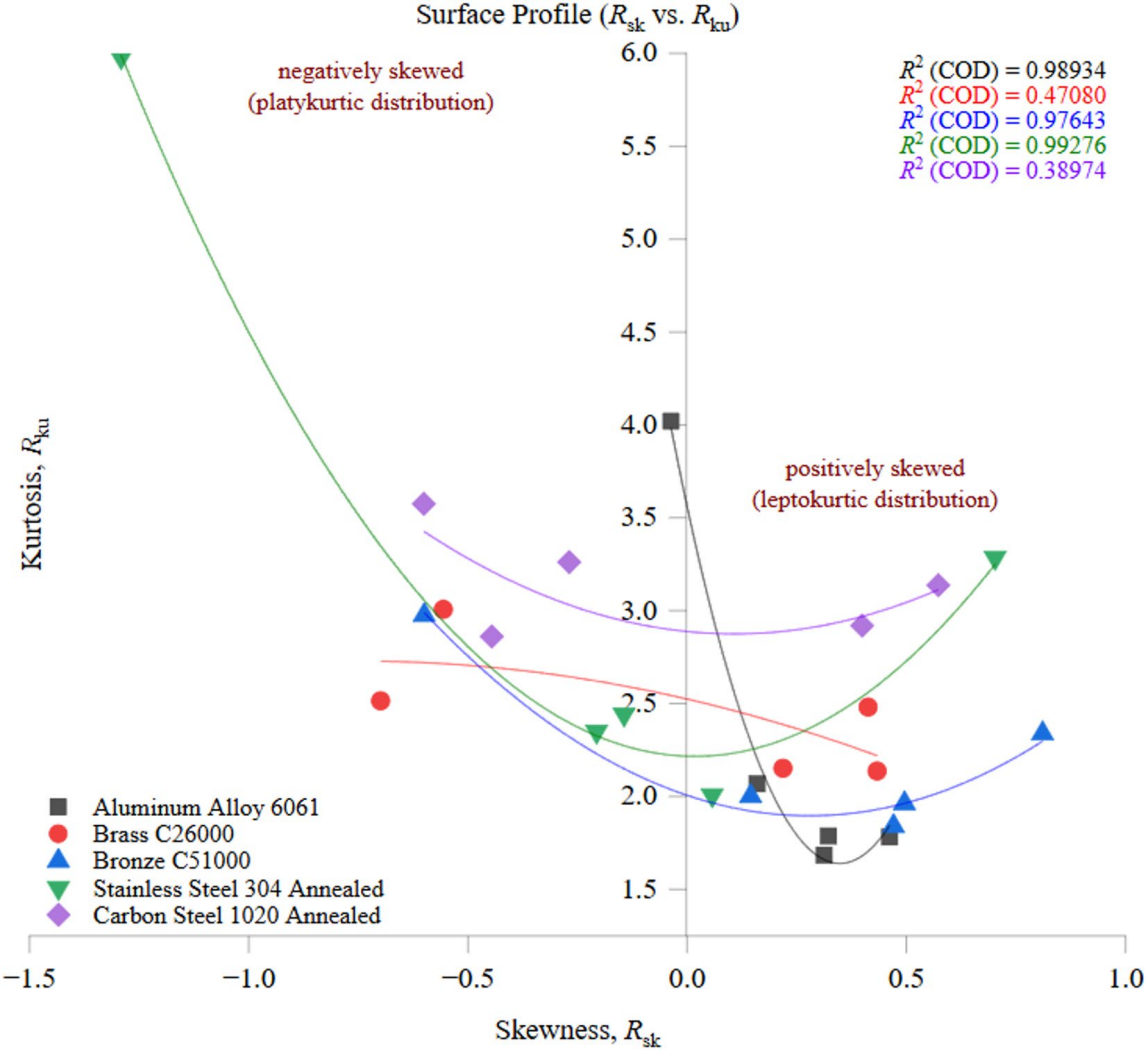
Surface Profile (*R*_sk_ *vs. R*_ku_).

Figure 15 presents a comparative analysis of surface skewness (*R*_sk_) and kurtosis (*R*_ku_) across the tested materials, highlighting distinct differences in surface texture characteristics influenced by material properties. These statistical parameters provide insights into the asymmetry and sharpness of surface irregularities, which are crucial for understanding material behavior in machining and post-processing applications. Aluminum alloy 6061 exhibited an *R*_sk_ value of approximately − 0.09 and an *R*_ku_ value of 2.39, indicating a surface profile slightly skewed toward valleys with a relatively uniform height distribution. The material’s high thermal conductivity (205 W/m·K) and low hardness (95 HBW) contribute to stable cutting conditions, preventing excessive tool wear and localized surface distortions. As a result, aluminum surfaces remain balanced and free from extreme peaks, minimizing wear on contact surfaces in tribological applications. The relatively low kurtosis value suggests a broad, well-distributed surface profile, indicative of a smooth and predictable texture.

Stainless Steel 304 displayed a more complex behavior, with an average *R*_sk_ value of −0.18 and an *R*_ku_ value of 3.21, signifying a slightly valley-dominated surface profile with sharper peaks than Aluminum. This outcome is driven by stainless steel’s low thermal conductivity (16 W/m·K) and high hardness (210 HBW), which increase cutting resistance and localized thermal stress. The higher kurtosis value suggests the presence of distinct peaks, possibly due to tool-workpiece interactions and the material’s strain-hardening effects, which contribute to uneven surface formations. These sharp features could negatively impact precision applications requiring uniform contact surfaces. Brass C26000 exhibited a slightly negative *R*_sk_ value (−0.38) and an *R*_ku_ value of 2.76, indicating a surface profile with deeper valleys and sharper features than Aluminum. These characteristics can be linked to Brass’s relatively high thermal conductivity (109 W/m·K) and moderate hardness (115 HBW), which facilitate efficient heat dissipation but allow for some localized machining irregularities. The negative skewness suggests that material removal during machining resulted in deeper depressions rather than raised peaks, which may affect lubricant retention in functional surfaces. Bronze C51000 followed a similar trend, with an *R*_sk_ value of 0.26 and an *R*_ku_ value of 2.22. These values indicate a surface skewed toward sharper peaks with a broad, less-defined height distribution. Bronze’s moderate thermal conductivity (60 W/m·K) and higher hardness (112.5 HBW) contribute to a more resistant cutting behavior, leading to pronounced peaks that may be influenced by tool deflection or chip adhesion during machining. The lower kurtosis value suggests a more uniform surface but with a tendency toward peak formation rather than valley dominance. Carbon Steel 1020 transitioned from slightly negative to neutral *R*_sk_ values (−0.07) and an *R*_ku_ value of 3.15, indicating a relatively balanced surface texture but with occasional sharp features under certain machining conditions. The material’s moderate hardness (150 HBW) and thermal conductivity (50 W/m·K) contribute to increased cutting resistance and heat accumulation, which can cause irregular material removal and surface imperfections. The elevated kurtosis suggests the presence of sharper peaks, likely due to tool wear effects and chip adhesion during prolonged cutting operations.

In summary, Fig. 15 highlights the crucial role of material properties in shaping surface texture characteristics. Softer, highly conductive materials like aluminum alloy 6061 exhibit more uniform and predictable surfaces with minimal sharpness, whereas harder, less thermally conductive materials like stainless steel 304 develop sharper peaks and irregular features due to increased cutting resistance and localized thermal effects. Brass and Bronze, which are intermediate materials, exhibit mixed behaviors that balance uniformity with moderate deviations in profile structure. These findings underscore the importance of selecting appropriate machining parameters to optimize surface topography, particularly for applications requiring consistent tribological and contact surface properties.

**Fig. 16:**
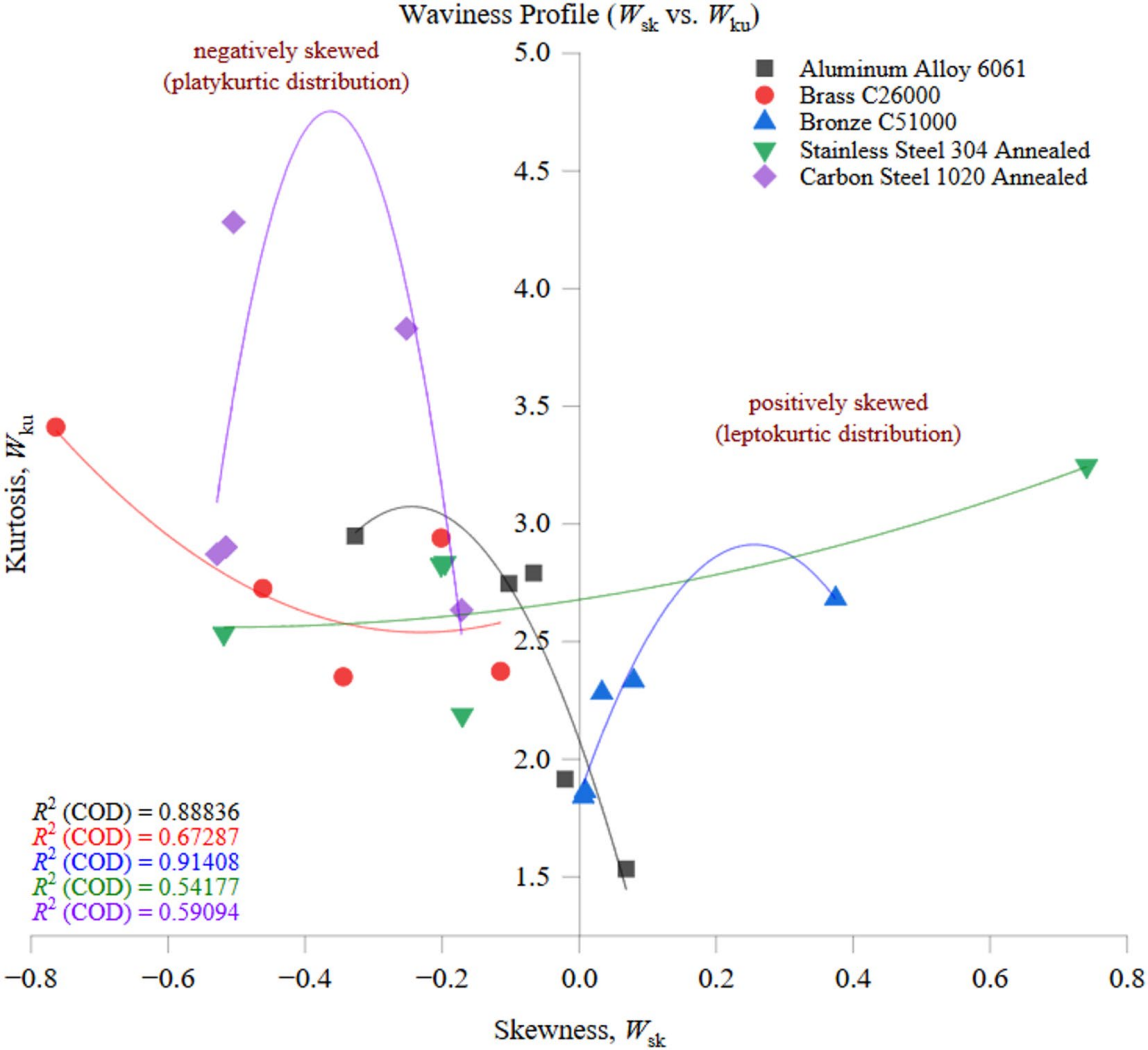
Waviness Profile (*W*_sk_ *vs. W*_ku_).

Figure 16 examines the relationship between waviness skewness (*W*_sk_) and kurtosis (*W*_ku_) across the tested materials, providing critical insights into the distribution and sharpness of surface undulations. These statistical parameters help characterize the formation of peaks and valleys in waviness profiles, which significantly impact functional surface properties such as load-bearing capacity, tribological behavior, and fatigue resistance. Aluminum alloy 6061 exhibited a *W*_sk_ value of approximately − 0.09 and a *W*_ku_ value of 2.39, indicating a nearly symmetrical waviness profile with minimal sharp peaks. These results reflect Aluminum’s superior thermal conductivity (205 W/m·K) and relatively low hardness (95 HBW), which ensure stable heat dissipation and minimal thermal stress during machining. Consequently, aluminum surfaces maintain a well-distributed undulation pattern, free from extreme irregularities. The slight deviation from perfect symmetry (negative skewness) suggests a tendency toward shallow valleys rather than pronounced peaks, which may enhance lubricant retention in tribological applications. Stainless Steel 304, in contrast, exhibited a more asymmetrical and peaked waviness profile, with *W*_sk_ values reaching approximately + 0.35 and *W*_ku_ values around 3.80. These values indicate a surface texture dominated by sharp peaks and irregular features due to stainless steel’s low thermal conductivity (16 W/m·K) and high hardness (210 HBW). The poor heat dissipation leads to localized thermal expansion and increased tool wear, resulting in waviness features with higher asperities. This trend highlights the greater difficulty in achieving uniform waviness in harder, less thermally conductive materials. Brass C26000 and Bronze C51000 demonstrated intermediate waviness skewness and kurtosis values, reflecting moderately asymmetric and sharp waviness distributions. Brass exhibited *W*_sk_ values of approximately − 0.15 and *W*_ku_ around 3.40, indicating a surface slightly skewed toward valleys with moderately sharp undulations. This behavior aligns with Brass’s high thermal conductivity (109 W/m·K) and moderate hardness (100 HBW), which help maintain a relatively stable cutting process but still allow for some variability in waviness. Bronze, with a *W*_sk_ value of approximately + 0.15 and *W*_ku_ around 3.30, showed a slight skew toward peaks. This result reflects Bronze’s moderate thermal conductivity (60 W/m·K) and hardness (112.5 HBW), which balance cutting stability and waviness variability. Moderate skewness and kurtosis suggest that both materials experience some waviness irregularities, though they are less extreme than those of stainless steel. Carbon steel 1020 displayed *W*_sk_ values transitioning from slightly negative to neutral, with *W*_ku_ values averaging around 3.30. These results indicate a surface texture with moderate asymmetry and sharpness, consistent with carbon steel’s thermal conductivity (50 W/m·K) and hardness (150 HBW). The material’s higher cutting resistance and moderate heat dissipation contribute to greater waviness, making its profile more irregular than Aluminum’s but more uniform than stainless steel’s. These factors highlight the importance of carefully controlled cutting conditions when machining carbon steel to minimize waviness fluctuations.

In summary, Fig. 16 highlights the critical influence of material properties on the symmetry and sharpness of waviness profiles. Softer, thermally conductive materials like Aluminum alloy 6061 exhibit smooth and symmetrical waviness distributions, minimizing surface irregularities. In contrast, harder, less thermally conductive materials like stainless steel 304 exhibit more pronounced peaks and greater asymmetry, requiring precise adjustments to machining parameters to reduce surface inconsistencies. The balance between surface smoothness and variability in intermediate materials, including Brass, Bronze, and Carbon Steel, necessitates careful material selection and process optimization to achieve superior surface quality in machining applications.

The study extensively uses statistical shape metrics, such as skewness (*R*_sk_, *W*_sk_) and kurtosis (*R*_ku_, *W*_ku_), which provide insights into peak–valley asymmetry and sharpness. These descriptors have practical implications for real-world applications: surfaces with high kurtosis may contain sharp asperities that concentrate mechanical stresses and initiate fatigue cracks, whereas negatively skewed surfaces may favor lubricant retention, enhancing tribological performance. Therefore, the shape-based texture metrics presented here are not just statistical abstractions; they provide predictive indicators of component performance under load, wear, or fluid interaction.

Beyond conventional skewness and kurtosis, 3D areal parameters (*S*_a_, *S*_q_, *S*_sk_, *S*_ku_) and fractal-based descriptors can capture multiscale surface anisotropy and functional properties such as load-bearing capacity and lubrication behavior^37^. While powerful, these methods were not incorporated here because the present study was deliberately scoped to ISO 4287 line-based descriptors to establish a clear, reproducible baseline for multi-material comparison under unified machining conditions. This ensures methodological alignment with widely adopted industrial standards, while avoiding an overly broad scope in a single study. Importantly, the present work should be viewed as a foundation for future research, where 3D surface topology and fractal analysis will be integrated as a natural extension. By building on the baseline relationships established here, the next phase of this research will aim to deliver a more comprehensive, multi-scale framework for predicting and controlling surface integrity in CNC turning.

## Conclusion

This study conducted a comparative analysis of surface integrity in CNC turning under identical, controlled dry machining conditions. Five commonly used engineering alloys (Aluminum Alloy 6061, Brass C26000, Bronze C51000, Carbon Steel 1020, and Stainless Steel 304) were examined to establish a reproducible baseline linking material properties, namely thermal conductivity and hardness, to surface roughness and waviness descriptors. Unlike many earlier works focused on single materials or purely parametric optimization, the present study adopted a property-driven, cross-material perspective to reveal how fundamental thermomechanical characteristics govern machining outcomes.

The results demonstrate that thermal conductivity strongly influences surface integrity in combination with material-specific deformation and chip-formation mechanisms. Aluminum Alloy 6061 exhibited relatively low mean *R*_a_ (~ 1.5 μm) but the highest variability (CV ≈ 82%) due to ductility-driven adhesion and built-up edge formation. Brass C26000 and Bronze C51000 achieved moderate mean *R*_a_ values (~ 1.2–1.6 μm) with lower variability (CV ≈ 35–42%). Carbon Steel 1020 produced consistently rough surfaces (mean *R*_a_ ≈ 4.2 μm, CV ≈ 27%), reflecting poor heat dissipation and wear-prone chip flow. In contrast, Stainless Steel 304 achieved the most uniform and smooth surfaces (mean *R*_a_ ≈ 0.9 μm, CV ≈ 18%) due to stable strain hardening and consistent curled chips. Advanced statistical descriptors supported these trends: profile ratios (*R*_q_/*R*_a_ ≈ 1.06–1.15, *W*_q_/*W*_a_ ≈ 1.05–1.12) revealed differences in surface uniformity, while skewness indicated valley-dominated surfaces for Stainless Steel 304 (*R*_sk_ ≈ − 0.25) and peak-dominated, wear-prone surfaces for Aluminum Alloy 6061 and Carbon Steel 1020 (*R*_sk_ ≈ + 0.32 and + 0.41). Sensitivity analysis showed that a ± 10% variation in thermal conductivity produces a 5.8–6.3% change in *R*_a_ across the five alloys. One-way ANOVA (*F*(4,145) = 205.83, *p* < 1.0 × 10⁻^58^) confirmed that material type accounts for over 84% of the variance in *R*_a_, and Tukey post-hoc tests verified that Stainless Steel 304 consistently produced significantly smoother surfaces than Carbon Steel 1020, Aluminum Alloy 6061, Brass C26000, and Bronze C51000, while Brass and Bronze occupied intermediate positions.

Several limitations are acknowledged. Tool wear was controlled through strict insert replacement but not quantitatively tracked, and chip morphology was qualitatively observed but not systematically documented. In addition, the analysis relied on ISO 4287 line-based descriptors, which provide only a partial description of surface complexity. Future work will incorporate quantitative flank-wear measurements, systematic chip-morphology imaging, microstructural characterization, 3D areal surface parameters, and direct thermal-conductivity measurements to enhance predictive capability. Overall, this study provides a reproducible baseline for surface integrity analysis in multi-material CNC turning and demonstrates that machining performance cannot be fully understood without a property-driven, cross-material perspective. The proposed framework offers a practical basis for material-aware process planning and quality control in precision CNC machining.

## Data Availability

The datasets used and/or analyzed during the current study available from the corresponding author on reasonable request.
